# Dynamic representation of music induced emotion regulation and neuroendocrine responses

**DOI:** 10.3389/fendo.2026.1863088

**Published:** 2026-09-14

**Authors:** Shuang Shi

**Affiliations:** Zhengzhou Technical College, Zhengzhou, China

**Keywords:** ComponentEnergyTemporal model, dynamic representation, modular structure learning, music induced emotion, neuroendocrine Responses

## Abstract

**Introduction:**

This study introduces a novel methodology for dynamically representing the regulation of music-induced emotions and associated neuroendocrine responses. The research addresses the limitations of traditional approaches, which often fail to capture the intricate and dynamic interactions between musical stimuli and the resulting emotional and physiological changes.

**Methods:**

To overcome these challenges, we propose the Componen tEnergy Temporal model, which integrates three key components: the Component Interaction Resolver, the Energy Conserving Adapter, and the Temporal Forecast Adapter. These components collectively provide a robust framework for analyzing the temporal and energetic dynamics of music-induced emotional and physiological responses. Furthermore, the methodology incorporates modular structure learning and latent dynamics modeling to enhance adaptability and precision, enabling the model to account for individual variability and complex system behaviors.

**Results and discussion:**

Experimental results demonstrate the model’s efficacy in accurately predicting and representing the dynamic interplay between music, emotion, and neuroendocrine activity, achieving significant improvements in predictive accuracy and interpretability compared to existing methods. The findings underscore the potential of this approach to advance research in music therapy, emotional regulation, and related fields, offering a powerful tool for exploring the mechanisms underlying music’s impact on human physiology and emotion. This work provides a foundation for future studies aimed at developing personalized interventions and therapeutic strategies leveraging music’s regulatory effects on emotional and physiological states.

## 1 Introduction

The dynamic representation of music-induced emotion regulation and neuroendocrine responses is a burgeoning field of research that holds significant promise for enhancing our understanding of the complex interplay between auditory stimuli and physiological processes. Music has long been recognized for its ability to evoke emotions, but its potential as a tool for emotion regulation and its impact on neuroendocrine responses is only beginning to be fully appreciated. Not only does music offer a noninvasive and accessible means of influencing emotional states, but it also has the potential to modulate neuroendocrine functions, which are critical for maintaining homeostasis and overall well being (1). Furthermore, understanding the mechanisms underlying music-induced emotion regulation can contribute to the development of therapeutic interventions for mood disorders and stress-related conditions (2). This research is not only necessary for advancing theoretical knowledge but also for its practical applications in clinical settings, where music therapy is increasingly being integrated into treatment protocols (3).

Early investigations into music-induced emotion regulation primarily relied on approaches that emphasized explicit modeling of musical features and their emotional associations. These methods often utilized structured frameworks to encode relationships between auditory stimuli and emotional states, offering a systematic way to analyze the interplay between music and emotions (4). While these approaches provided valuable insights, they were constrained by their inability to adapt to the variability of individual emotional experiences and the intricate nature of neuroendocrine responses (5). The oversimplification of physiological processes further limited their applicability, underscoring the need for more flexible and comprehensive methodologies (6).

To overcome these limitations, researchers began employing computational techniques capable of identifying patterns and relationships within large datasets. By analyzing extensive collections of musical and physiological data, these methods enabled the discovery of complex associations that were previously unattainable (7). Algorithms such as decision trees and support vector machines demonstrated improved accuracy in predicting emotional responses, offering a more scalable approach to studying music-induced emotion regulation (8). However, these models often struggled to provide insights into the underlying mechanisms and required substantial effort in feature selection, which could limit their generalizability across diverse contexts.

The emergence of advanced neural network architectures has further transformed the field by enabling end to end modeling of emotional and physiological responses to music. Techniques such as convolutional and recurrent neural networks have proven effective in capturing both the hierarchical structure of musical features and their temporal dynamics (9). Pre trained models have also facilitated the generalization of findings across different musical genres and emotional contexts, significantly enhancing the robustness of these approaches (10). Despite these advancements, challenges remain, including the high computational demands and limited interpretability of these models, which can hinder their practical application in clinical and real world settings (11).

Based on the limitations of the aforementioned approaches, the ComponentEnergyTemporal model is proposed for dynamic representation of music induced emotion regulation and latent neuroendocrine responses. Different from generic hybrid AI frameworks that mainly combine symbolic representation, statistical learning, and neural modeling through broad feature fusion, ComponentEnergyTemporal is designed around the specific temporal and component level structure of music induced affective dynamics. The model decomposes musical stimuli into interpretable component streams, models pairwise interactions among tempo, rhythm, melody, and harmony related patterns, regulates dynamic response evolution through a latent energy potential, and captures temporal affective transitions through recurrent forecasting. This design enables the model to represent the dynamic interplay between auditory stimuli, emotional states, and latent physiological responses in a more structured and interpretable manner. The advantage of this formulation lies in its task aligned integration: component interaction modeling improves interpretability, latent energy potential regulation supports stable dynamic representation, and temporal forecasting captures accumulated musical context over time. Therefore, the proposed model extends existing hybrid AI ideas from general feature integration to a specialized dynamic framework for music induced emotional regulation.

The main contributions of this study are summarized as follows:

- A ComponentEnergyTemporal model is proposed for dynamic representation of music induced emotional regulation and latent neuroendocrine responses. Compared with current hybrid AI frameworks, the proposed model is not limited to general multimethod fusion, but explicitly integrates component interaction modeling, latent energy potential regulation, and temporal forecasting within one task specific dynamic architecture.
- A Component Interaction Resolver is introduced to model structured relations among tempo, rhythm, melody, and harmony related musical components. This module provides a component level interpretation of how musical patterns jointly contribute to emotion prediction, offering clearer interpretability than conventional global feature fusion strategies.
- A latent energy potential regulation mechanism is developed to stabilize affective response dynamics. The energy term is defined as a model internal activation potential rather than a physical or biochemical energy quantity, allowing the model to represent stimulus driven excitation and dissipative recovery in a biologically more plausible way.
- A Temporal Forecast Adapter is designed to capture the sequential evolution of music induced emotional states. This module enables the model to use accumulated musical context across segments rather than treating each audio segment as an isolated observation.
- Experiments on PMEmo and EMOPIA demonstrate that the proposed model improves four quadrant music emotion classification over traditional learning, convolutional, recurrent, pretrained audio, Transformer based, and lightweight neural baselines, while maintaining a favorable accuracy efficiency tradeoff.

## 2 Related work

### 2.1 Neuroendocrine mechanisms in music emotion

The investigation into neuroendocrine mechanisms underlying music-induced emotional regulation has revealed significant insights into how hormonal responses are modulated by musical stimuli. The neuroendocrine system, encompassing the hypothalamus, pituitary gland, and adrenal glands, orchestrates the secretion of hormones such as cortisol, oxytocin, and adrenaline, which are integral to emotional and stress-related processes (9). Studies have demonstrated that listening to music can influence cortisol levels, with relaxing music often associated with reductions in cortisol, thereby alleviating stress and promoting relaxation (12). Conversely, stimulating music has been observed to elevate cortisol levels, potentially enhancing alertness and arousal (10). Oxytocin, a hormone linked to social bonding and emotional intimacy, has also been shown to increase during musical activities, particularly in group settings such as choirs or ensembles, fostering a sense of community and emotional connection (13). Adrenaline, a key component of the fight or flight response, is similarly affected by music, with fast paced compositions eliciting heightened arousal and energy, while slower, calming music reduces adrenaline levels, promoting relaxation (11). Individual differences, including musical preferences and personality traits, further modulate these neuroendocrine responses, with familiar and preferred music eliciting stronger emotional and physiological effects (14). The therapeutic implications of these findings are profound, as music therapy can leverage these hormonal pathways to address conditions such as anxiety, depression, and stressrelated disorders (15). By tailoring interventions to specific neuroendocrine mechanisms, practitioners can optimize the efficacy of music based treatments (16). The interplay between music, emotion, and hormonal activity underscores the complexity of neuroendocrine responses and their potential applications in emotional regulation (17).

### 2.2 Neural correlates of music emotion

The neural correlates of music-induced emotional experiences have been extensively studied, revealing the intricate interplay between brain regions and networks involved in processing musical stimuli. Neuroimaging techniques, including functional magnetic resonance imaging and electroencephalography, have identified key areas such as the limbic system, prefrontal cortex, and auditory cortex as central to music-induced emotional responses (18). The limbic system, comprising structures like the amygdala, hippocampus, and nucleus accumbens, is particularly significant, with the amygdala activated by emotionally charged music, reflecting its role in processing pleasure and fear (19). The hippocampus, associated with memory and emotional regulation, is engaged during music listening, especially when music evokes autobiographical memories or nostalgia (20). The nucleus accumbens, a critical component of the brain’s reward system, is activated by pleasurable music, often accompanied by dopamine release, highlighting the rewarding nature of musical experiences (21). The prefrontal cortex, responsible for higher order cognitive functions and emotional regulation, exhibits increased activity during music listening, particularly when complex emotional experiences are involved (22). The auditory cortex, while primarily tasked with sound processing, interacts with other brain regions to contribute to the emotional impact of music, emphasizing the interconnectedness of neural networks (23). Individual differences, such as musical training and personality traits, further influence neural responses, with musicians often displaying enhanced sensitivity to musical nuances and emotional content (24). Understanding these neural mechanisms has significant implications for therapeutic applications, including music therapy and neurofeedback, which aim to leverage brain activity modulation to promote emotional well being (25). By identifying the neural pathways involved in music-induced emotions, researchers and clinicians can develop targeted interventions to enhance emotional regulation and resilience (26). The complexity of neural responses to music underscores the profound impact of musical stimuli on emotional and cognitive processes (27).

### 2.3 Cultural influences on music emotion

The role of cultural influences in shaping music-induced emotional experiences is a critical area of research, highlighting the interplay between cultural norms, values, and social contexts in modulating emotional responses to music. Cultural differences in musical preferences are evident, with Western traditions often emphasizing harmonic and melodic structures, while other cultures may prioritize rhythm and timbre, reflecting diverse aesthetic values and emotional interpretations (28). Familiarity with culturally relevant music enhances emotional resonance, as exposure and socialization shape individual preferences and responses (4). Emotional expression in music is similarly influenced by cultural norms, with some societies valuing emotional restraint and others encouraging expressiveness, leading to varying intensities of emotional reactions (12). The social functions of music, such as its role in rituals, celebrations, and communal activities, further underscore its cultural significance, fostering social cohesion and emotional bonding through shared musical experiences (10). Group music making activities, including traditional dances and choral singing, exemplify how music promotes social harmony and emotional intimacy within cultural contexts (13). The interpretation of musical emotions also varies across cultures, with certain musical elements conveying distinct emotional meanings depending on cultural background (11). Globalization and cultural exchange have introduced hybrid forms of musical expression, blending traditions and creating novel emotional experiences that reflect the dynamic nature of cultural influences on music (14). These insights have practical applications in multicultural music therapy, where culturally sensitive interventions can address the emotional needs of diverse populations (15). Cross cultural communication benefits from understanding how music bridges cultural divides, fostering empathy and mutual understanding through shared emotional experiences (16). The study of cultural influences on music-induced emotion regulation underscores the importance of considering cultural diversity in both research and therapeutic practices (17).

### 2.4 Graph structured dynamic modeling

Graph structured dynamic modeling provides a broader computational perspective for representing interacting components in time varying systems. Although this line of research is not specific to music induced emotion regulation or neuroendocrine response analysis, it is methodologically relevant to component interaction modeling, temporal dependency learning, and latent dynamics. Spatio temporal graph attention networks have been used to model non stationary systems by combining temporal evolution with graph based relational learning. For example, FSTGAT integrates temporal modeling and graph attention mechanisms to capture dynamic dependencies in financial systems, showing how time varying interactions can be represented through structured attention over relational components (29). In graph theoretic studies, edge connectivity analysis of expanded *k* ary *n* cubes provides insight into robustness and connectivity properties of structured networks (30). Directed graph studies on ordered and Hamilton digraphs further highlight the role of path based connectivity and directed interaction structures (31). These studies are not direct models of music induced physiological regulation, but they support the broader motivation for representing musical components, emotional states, and latent physiological variables as an interaction network with temporal dependencies.

## 3 Methods

### 3.1 Overview

This section outlines the proposed methodology for modeling the dynamic interactions between musicinduced emotional regulation and neuroendocrine responses. The approach is designed to systematically capture the complex relationships between musical stimuli, emotional states, and physiological changes, employing advanced computational techniques to establish a rigorous analytical framework. The methodology is divided into distinct components, each addressing a specific aspect of the problem, and is structured to ensure both theoretical robustness and practical applicability.

The foundational concepts and mathematical formulations are introduced in subsection 3.2, where the interplay between music, emotion, and neuroendocrine responses is formalized. This subsection defines the theoretical constructs that describe the influence of musical stimuli on emotional states and the associated neuroendocrine dynamics. Key variables and parameters are introduced, including the emotional state vector **e**(*t*), the neuroendocrine response vector n(*t*), and the musical feature vector m(*t*), which collectively form the basis for subsequent modeling efforts. The relationships between these variables are expressed through a set of governing equations, which serve as the foundation for the development of the proposed model.

Subsection 3.3 introduces the ComponentEnergyTemporal model, a novel framework designed to encapsulate the temporal and interactive nature of music-induced emotional and physiological changes. The model is composed of three interconnected modules: the Component Interaction Resolver, the Energy Conserving Adapter, and the Temporal Forecast Adapter. The Component Interaction Resolver quantifies the influence of individual musical components on emotional states, represented as a mapping **e**(*t*) = *f*(m(*t*),w), where w denotes the learned interaction weights. The Energy Conserving Adapter enforces constraints to ensure that emotional transitions adhere to energy conservation principles, modeled as $\Delta E=\int_{t_{1}}^{t_{2}}P\left(t\right)dt$, where *P*(*t*) represents the emotional energy flux. The Temporal Forecast Adapter predicts future states **e**(*t* + Δ*t*) and n(*t* + Δ*t*) based on historical data, leveraging recurrent structures to capture temporal dependencies.

Subsection 3.4 details the modular structure learning and latent dynamics modeling strategy, which enhances the adaptability and precision of the ComponentEnergyTemporal model. Modular structure learning enables the integration of new data streams and evolving insights, allowing the model to remain flexible and responsive to changes in the underlying data distribution. Latent dynamics modeling uncovers hidden patterns within the data by introducing latent variables z(*t*), which are inferred through a variational framework. These latent variables capture unobserved factors that mediate the relationship between music, emotion, and neuroendocrine responses, providing a deeper understanding of the underlying processes.

The proposed methodology offers a comprehensive framework for analyzing the dynamic interactions between music, emotion, and physiology. By addressing each component of the problem with precision and rigor, this approach not only advances theoretical understanding but also provides practical tools for applications in music therapy, emotional regulation, and related fields.

### 3.2 Preliminaries

This section formalizes the problem of modeling the dynamic interactions between music-induced emotional regulation and neuroendocrine responses. The proposed framework integrates music stimuli with neuroendocrine feedback mechanisms to capture the complex interplay underlying emotional and physiological responses. The problem is formulated in a multi-dimensional space, where each dimension corresponds to a distinct component of the emotional and physiological response to music.

Let ℳ represent the set of music stimuli, with each stimulus m∈ℳ characterized by a feature vector ${\text{f}}_{m}\in {\mathbb{R}}^{n}$. The feature vector f*_m_* encodes properties of the music, such as tempo, rhythm, melody, and harmony. The emotional state elicited by a music stimulus is represented as a vector $e\in {\mathbb{R}}^{k}$, where each component of **e** corresponds to a specific emotional dimension, such as arousal or valence.

The neuroendocrine response is modeled as a vector $\text{n}\in {\mathbb{R}}^{l}$, which encapsulates the levels of various hormones and neurotransmitters influenced by the emotional state. In the empirical setting of this study, n is treated as a latent physiological response variable rather than a directly observed hormonal assay sequence, because the adopted public datasets provide music audio and emotion annotations but do not provide synchronized cortisol, oxytocin, adrenaline, or dopamine measurements. The relationship between the music features, emotional state, and neuroendocrine response is described by a mapping $\Phi :{\mathbb{R}}^{n}\to {\mathbb{R}}^{k}\times {\mathbb{R}}^{l}$, which is the target of the learning process.

The temporal dynamics of these interactions are critical, as the effects of music on emotional and neuroendocrine states evolve over time. A discrete time index $t\in {\mathbb{Z}}^{+}$ is introduced to represent time steps, and the state of the system at time *t* is defined as s*_t_* = (e*_t_*,n*_t_*). The time index is defined at the segment level rather than at the acoustic frame level. Each music clip is divided into fixed length 10 second segments with a stride of 5 seconds during preprocessing. Therefore, f*_m_*(*t*) denotes the acoustic feature representation summarized within the *t* th segment, rather than an instantaneous frame level descriptor. This definition provides a coarser temporal unit for modeling affective evolution and reduces the mismatch between rapidly varying acoustic events and slower physiological regulation.

Because emotional reactions and neuroendocrine responses may evolve at different time scales, the coupling between e*_t_* and n*_t_* is modeled with a segment level delay. Let *τ_n_* denote the neuroendocrine delay measured in segment steps. The delayed term reflects the fact that measurable hormonal responses usually emerge more slowly than short term acoustic or emotional fluctuations. The evolution of the system is governed by the following delayed coupled differential equations Equations 1, 2:

(1) $$\frac{de_{t}}{dt}=\text{A}{\text{e}}_{t}+\text{B}{\text{f}}_{m}\left(t\right)+\text{C}{\text{n}}_{t-\tau_{n}},$$

(2) $$\frac{dn_{t}}{dt}=\text{D}{\text{n}}_{t}+\text{E}{\text{e}}_{t-\tau_{n}},$$

where $\text{A}\in {\mathbb{R}}^{k\times k}$, $\text{B}\in {\mathbb{R}}^{k\times n}$, $\text{C}\in {\mathbb{R}}^{k\times l}$, $\text{D}\in {\mathbb{R}}^{l\times l}$, and $\text{E}\in {\mathbb{R}}^{l\times k}$ are parameter matrices. The matrix A captures the intrinsic dynamics of the emotional state, while B quantifies the influence of music features on emotion. The feedback from the neuroendocrine system to the emotional state is modeled by C. The matrices D and **E** describe the dynamics of the neuroendocrine response and its delayed dependence on the emotional state, respectively. The delay parameter *τ_n_* provides a simple multi scale approximation for the slower biological response process. When direct neuroendocrine measurements are unavailable, *τ_n_* is treated as a latency parameter in the latent physiological dynamics rather than as an observed hormone secretion time. This formulation does not assume instantaneous hormonal changes and allows the model to represent delayed physiological feedback at the segment level.

To account for unobserved factors influencing the system, a latent variable model is introduced. The observed state **s***_t_* is assumed to be influenced by latent variables $z_{t}\in {\mathbb{R}}^{p}$, with the relationship expressed as Equation 3:

(3) $$s_{t}=\psi_{s}\left(\text{F}{\text{z}}_{t}\right)+\in_{t},$$

where $\text{F}\in {\mathbb{R}}^{\left(k+l\right)\times p}$ is a mapping matrix, $\psi_{s}\left(\cdot \right)$ is a nonlinear observation function, and $\in_{t}\in {\mathbb{R}}^{k+l}$ is a noise term accounting for measurement errors and unmodeled dynamics. The nonlinear observation function allows the latent state to influence emotional and physiological responses through a flexible mapping rather than a purely linear projection. This design is useful when the observed response contains threshold effects, saturation effects, or subject dependent variability.

The latent variables evolve according to a stochastic nonlinear process Equation 4:

(4) $$z_{t+1}=\psi_{z}\left(Gz_{t}+Hu_{t}+Re_{t-\tau_{n}}+Qn_{t-\tau_{n}}\right)+\eta_{t},$$

where $G\in {\mathbb{R}}^{p\times p}$ is the latent transition matrix, $H\in {\mathbb{R}}^{p\times q}$ represents the influence of control inputs $u_{t}\in {\mathbb{R}}^{q}$, $R\in {\mathbb{R}}^{p\times k}$ and $Q\;in\;{\mathbb{R}}^{p\times l}$ project delayed emotional and neuroendocrine states into the latent space, $\psi_{z}\left(\cdot \right)$ is a nonlinear transition function, and $\eta_{t}\in {\mathbb{R}}^{p}$ is a process noise term. The delayed terms $e_{t-\tau_{n}}$ and $n_{t-\tau_{n}}$ enable the latent dynamics to represent slower physiological regulation and delayed feedback effects. The nonlinear transition function $\psi_{z}\left(\cdot \right)$ provides a compact approximation of nonlinearity in affective and neuroendocrine regulation, including bounded response intensity and state dependent transitions.

The objective is to estimate the parameters of the model, including the matrices A, B, C, D, E, F, G, H, R, and Q the nonlinear transition functions, and the noise covariances. This estimation aims to capture the temporal dynamics of music induced emotional regulation and latent neuroendocrine responses. In the experiments, the learning objective is supervised four quadrant music emotion classification because the adopted public datasets provide music audio and emotion annotations but do not provide synchronized hormone assay measurements. Therefore, the neuroendocrine state is inferred as a latent physiological response variable rather than learned from direct cortisol, oxytocin, adrenaline, or dopamine labels. The supervised loss is applied to the observed emotion labels, while the latent dynamics are regularized through the temporal forecasting objective and stochastic latent state modeling. This formulation supports dynamic representation of music induced emotional responses without assuming direct availability of measured endocrine sequences.

The proposed formulation treats music induced emotion and physiological regulation as a coupled dynamic process rather than a static mapping from acoustic features to emotion labels. Emotional responses evolve under the joint influence of current musical stimulation, accumulated affective context, intrinsic emotional persistence, and slower physiological feedback. The emotional state equation therefore represents internal state evolution, external musical input, and delayed physiological influence. The neuroendocrine formulation is biologically motivated by the slower time scale of physiological regulation relative to auditory perception and immediate emotional appraisal. The delay parameter provides a coarse segment level approximation of this difference rather than an exact hormone secretion interval. Since PMEmo and EMOPIA do not contain synchronized hormone measurements, the neuroendocrine state is interpreted as a latent physiological response variable. The Component Interaction Resolver is motivated by the interdependent effects of tempo, rhythm, melody, and harmony. These factors rarely influence emotion independently, and pairwise interaction modeling can capture how one component modifies the contribution of another. The component relations can also be interpreted as a data dependent graph, in which musical components are nodes and learned interactions are weighted edges. This view provides a structured basis for relation modeling and improves component level interpretability. The latent energy state is defined as a stability related activation potential rather than a physical or biochemical energy quantity. The excitation term represents increased activation caused by salient musical interactions, while the dissipative term represents recovery toward a baseline state. The Temporal Forecast Adapter further captures the cumulative and context dependent nature of music induced emotion by retaining information from preceding segments and predicting subsequent affective states. The Component Interaction Resolver models structured musical relations, the Energy Potential Adapter constrains temporal stability, and the Temporal Forecast Adapter captures sequential dependence. The latent physiological state and delay mechanism provide a biologically inspired approximation of slower regulatory feedback without claiming to reproduce the complete human neuroendocrine system.

### 3.3 ComponentEnergyTemporal

In this section, we introduce our novel model, ComponentEnergyTemporal, designed to dynamically represent music-induced emotion regulation and neuroendocrine responses. The model is structured to capture the intricate interactions between musical components and their temporal evolution, while conserving the energy dynamics inherent in emotional and neuroendocrine processes. The ComponentEnergyTemporal model is composed of three primary modules, each representing a distinct innovation: Interaction Quantification and Aggregation, Energy Conservation Dynamics, and Temporal Dependency Modeling. These modules collectively ensure the model’s ability to accurately represent and predict the complex dynamics of music-induced emotional and neuroendocrine responses.

As shown in **Figure 1**, the proposed framework contains three sequential modules: the Component Interaction Resolver, the Energy Potential Adapter, and the Temporal Forecast Adapter. The figure presents the complete computational workflow from spectrogram encoding and musical component interaction modeling to latent energy regulation, temporal forecasting, and four quadrant emotion classification.

**Figure 1:**
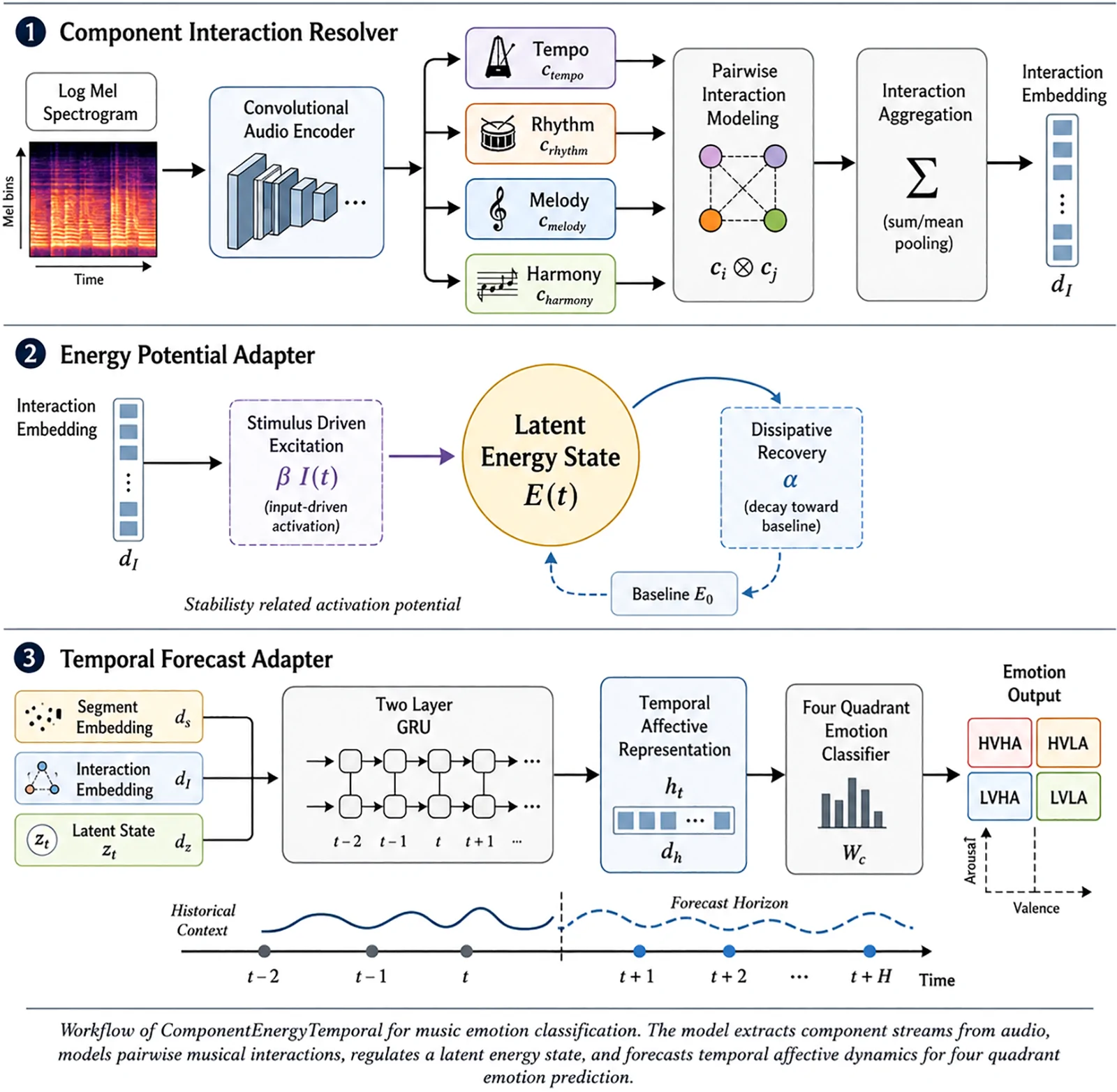
Detailed architecture and workflow of the proposed ComponentEnergyTemporal model. The Component Interaction Resolver first converts the log Mel spectrogram into a segment representation through a convolutional audio encoder and constructs four component streams corresponding to tempo, rhythm, melody, and harmony. Pairwise outer product interactions are then computed and aggregated to obtain the interaction embedding. The Energy Potential Adapter uses the interaction embedding to regulate a latent energy state through stimulus driven excitation and dissipative recovery toward the baseline potential. The Temporal Forecast Adapter combines the segment embedding, interaction embedding, and latent state through a two layer gated recurrent unit to generate a temporal affective representation. The final classifier predicts one of the four valence arousal emotion quadrants. The figure illustrates the computational architecture implemented in the experiments and does not represent direct hormone measurement.

Interaction Quantification and Aggregation: The first innovation focuses on identifying and quantifying the interactions between different musical components. As shown in **Figure 2**, the Interaction Quantification and Aggregation module first decomposes the segment embedding into tempo, rhythm, melody, and harmony related component streams. It then models pairwise relations among these components and aggregates the resulting interaction representations into a unified interaction embedding. Let $C=\left\{c_{1},c_{2},\ldots ,c_{n}\right\}$ represent the set of musical components, where each *c_i_* is a vector capturing the features of the *i*-th component. The interaction between components *c_i_* and *c_j_* is modeled as Equation 5:

**Figure 2:**
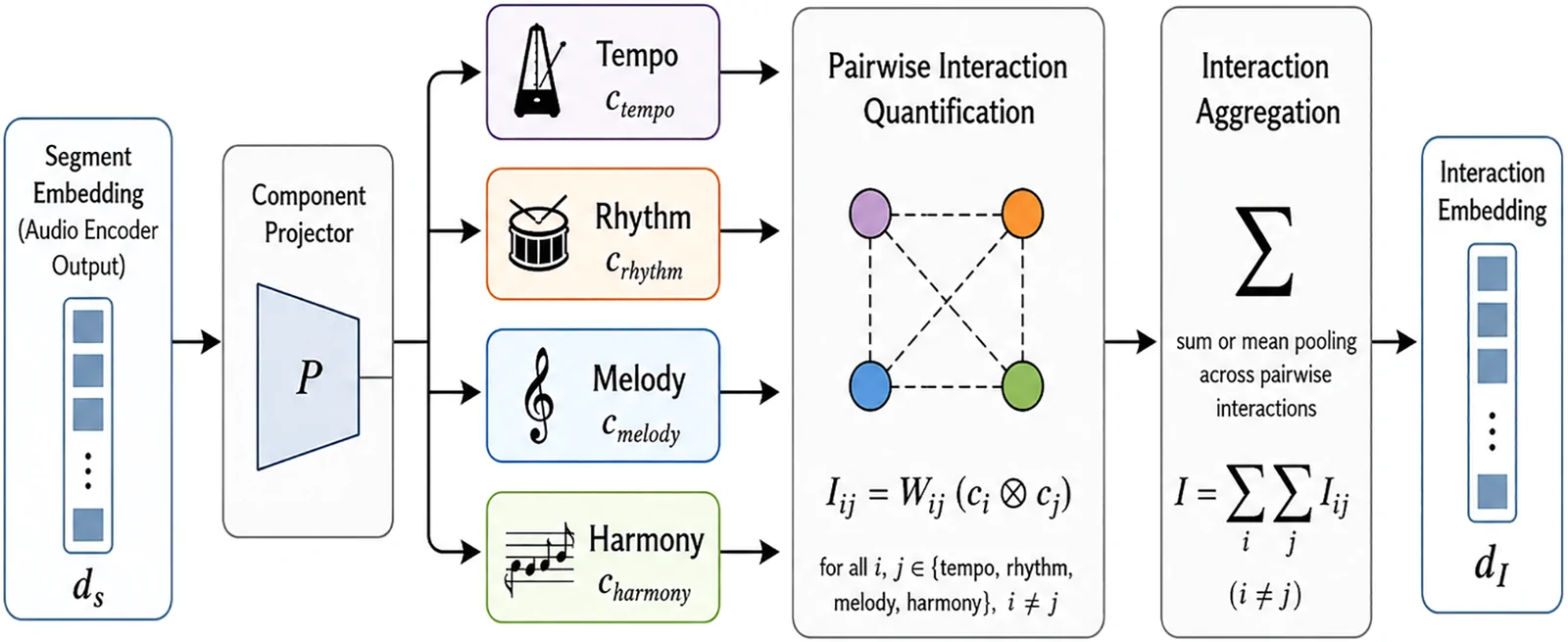
Detailed workflow of the interaction quantification and aggregation module. The segment embedding produced by the audio encoder is projected into four component streams corresponding to tempo, rhythm, melody, and harmony. Pairwise interactions are then quantified through learnable outer product based mappings between component pairs. The resulting interaction representations are aggregated by sum or mean pooling to obtain a compact interaction embedding for subsequent latent energy regulation and temporal affective modeling.

(5) $$I\left(c_{i},c_{j}\right)=W_{ij}\cdot \left(c_{i}⊗c_{j}\right)$$

where W*_ij_* is a learned weight matrix and ⊗ denotes the outer product. The interactions are aggregated to form a comprehensive interaction matrix **I** Equation 6:

(6) $$I=\sum_{i=1}^{n}\sum_{j=1}^{n}I\left(c_{i},c_{j}\right)$$

This matrix **I** serves as the foundation for subsequent modules by encapsulating the relationships between musical components. The quantification of these interactions allows the model to capture the nuanced interplay between components, which is critical for understanding their combined effects on emotional and neuroendocrine responses.

Dissipative Energy Potential Dynamics: The second innovation introduces a latent energy potential to regulate the temporal stability of emotional and neuroendocrine response dynamics. Here, energy does not denote physical energy, thermodynamic energy, acoustic energy, or biochemical energy. Instead, it represents a model internal activation potential that summarizes the intensity of music induced affective and latent physiological responses. This potential is used as a stability related quantity, similar to a Lyapunov style potential, rather than as a directly measured biological variable.

Let ℰ(t) represent the latent energy potential at time *t*, and let E_0_ denote the baseline potential. The potential evolves through a dissipative process driven by musical component interactions Equation 7:

(7) $$\frac{dℰ\left(t\right)}{dt}=-\alpha \left(ℰ\left(t\right)-{ℰ}_{0}\right)+\beta \sum_{i=1}^{n}\sum_{j=1}^{n}I\left(c_{i},c_{j}\right)$$

where *α >* 0 controls the rate of return toward the baseline potential, and *β >* 0 controls the contribution of musical component interactions to the latent activation potential. The first term represents dissipative recovery, while the second term represents stimulus driven excitation from component interactions. The solution to this equation is given by Equation 8:

(8) $$ℰ\left(t\right)={ℰ}_{0}+\left(ℰ\left(0\right)-{ℰ}_{0}\right)e^{-\alpha t}+\beta \int_{0}^{t}e^{-\alpha \left(t-\tau \right)}\sum_{i=1}^{n}\sum_{j=1}^{n}I\left(c_{i},c_{j}\right)\;d\tau$$

This formulation does not assume that musical input energy is physically conserved or directly converted into emotional or endocrine output energy. Human emotional and neuroendocrine responses are treated as open and dissipative processes. A strong interaction contribution can temporarily increase the latent activation potential, while the dissipative term prevents unbounded growth and encourages gradual recovery toward the baseline. By incorporating the interaction matrix **I**, the latent energy potential is directly influenced by quantified musical component interactions, providing a stability regularizer for modeling the temporal evolution of music induced emotional and latent physiological states.

Temporal Dependency Modeling: The third innovation is designed to predict future states of the system by capturing the temporal dependencies in the data. This is achieved using a recurrent neural network (RNN) framework, where the hidden state h*_t_* at time *t* is updated as Equation 9:

(9) $${\text{h}}_{t}=\sigma \left(\text{U}{\text{h}}_{t-1}+\text{V}{\text{I}}_{t}+\text{b}\right)$$

where *σ* is an activation function, **U** and **V** are weight matrices, and **b** is a bias vector. The output of the RNN, representing the predicted state, is given by Equation 10:

(10) $${\text{o}}_{t}={\text{W}}_{o}{\text{h}}_{t}+{\text{b}}_{o}$$

where W*_o_* and b*_o_* are the output weight matrix and bias, respectively. This module leverages the temporal structure of the data to forecast the evolution of emotional and neuroendocrine responses, providing insights into their long term dynamics.

By integrating these three innovations, the ComponentEnergyTemporal model provides a comprehensive framework for understanding and predicting the dynamic interplay between music, emotion, and neuroendocrine responses. This model not only captures the immediate effects of musical components but also accounts for their long term impact through energy conservation and temporal forecasting.

### 3.4 Modular structure learning and latent dynamics modeling

In this subsection, we delve into the innovative strategies employed to enhance the dynamic representation of music-induced emotion regulation and neuroendocrine responses. Our approach is grounded in three pivotal strategies: Component Interaction Resolution, Energy Conservation Dynamics, and Temporal Forecasting of Emotional States. These strategies are meticulously designed to address the complexities inherent in the domain of music-induced emotional and physiological responses, leveraging the capabilities of the ComponentEnergyTemporal model. As shown in **Figure 3**, the proposed framework integrates component interaction learning, latent energy regulation, and recurrent temporal forecasting within a unified modular architecture.

**Figure 3:**
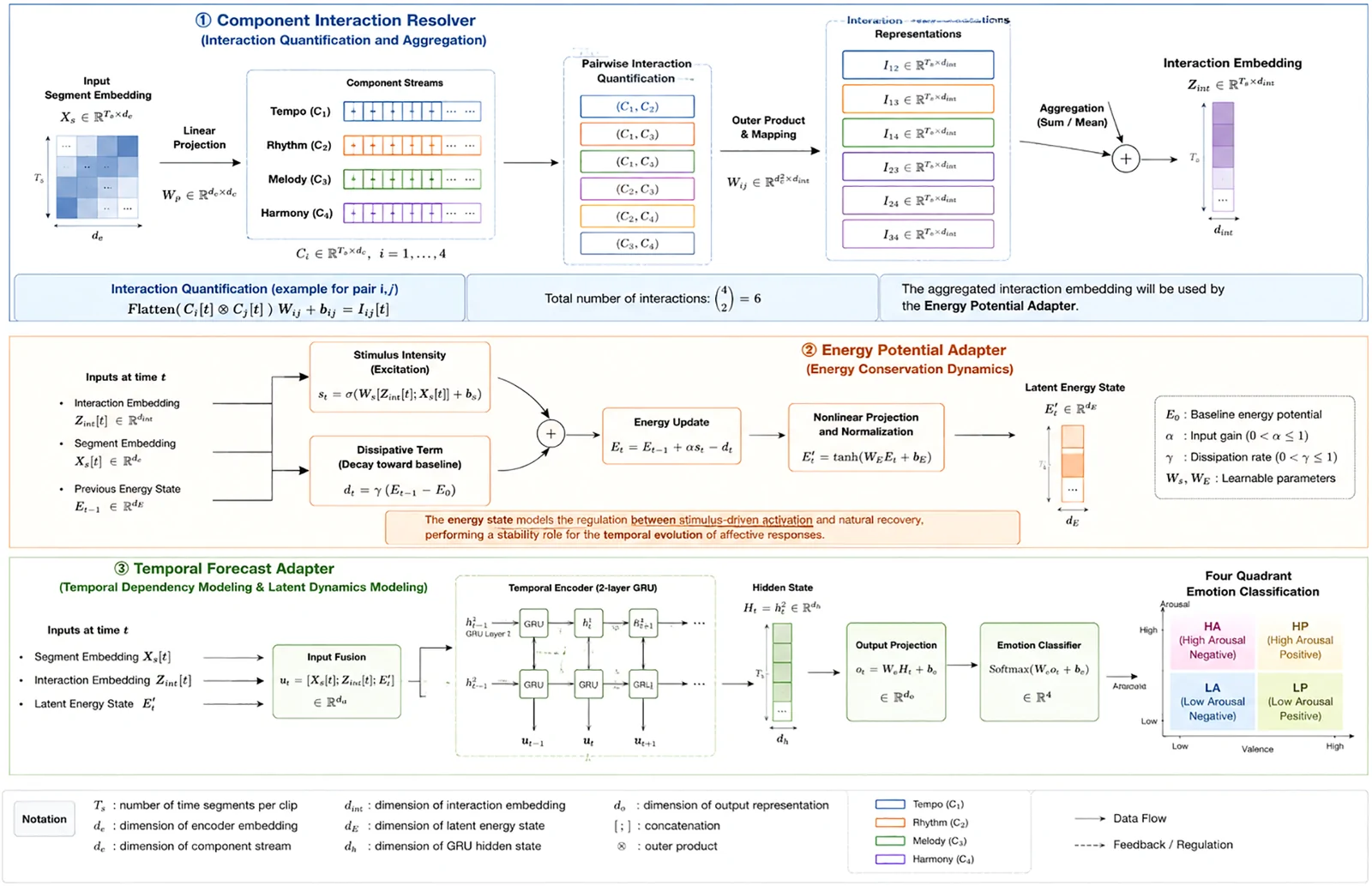
Detailed architecture of the modular structure learning and latent dynamics modeling framework. The Component Interaction Resolver first projects the segment embedding into tempo, rhythm, melody, and harmony related component streams, quantifies all pairwise interactions through outer product based mappings, and aggregates them into a compact interaction embedding. The Energy Potential Adapter then combines the interaction embedding, segment representation, and previous energy state to model stimulus driven excitation and dissipative recovery toward a baseline, producing a latent energy state for temporal regulation. The Temporal Forecast Adapter fuses the segment embedding, interaction embedding, and latent energy state, and processes the resulting sequence with a two layer gated recurrent unit. The temporal representation is finally projected into four quadrant emotion probabilities in the valence arousal space. Solid arrows indicate the main data flow, while dashed arrows indicate regulatory or feedback relations.

Component Interaction Resolution: This strategy is predicated on the notion of decomposing the intricate interactions between musical components and emotional responses into manageable modules. This decomposition allows for a more granular analysis and understanding of the underlying processes. The Component Interaction Resolver module plays a crucial role in this strategy by systematically identifying and resolving the interactions between various musical components and their corresponding emotional effects. This module employs advanced techniques to parse the complex relationships, ensuring that each component’s influence is accurately captured and represented. As shown in **Figure 4**, the Component Interaction Resolution module decomposes each segment embedding into four musical component streams, quantifies all pairwise relations, and aggregates them into a compact interaction representation for downstream modeling.

**Figure 4:**
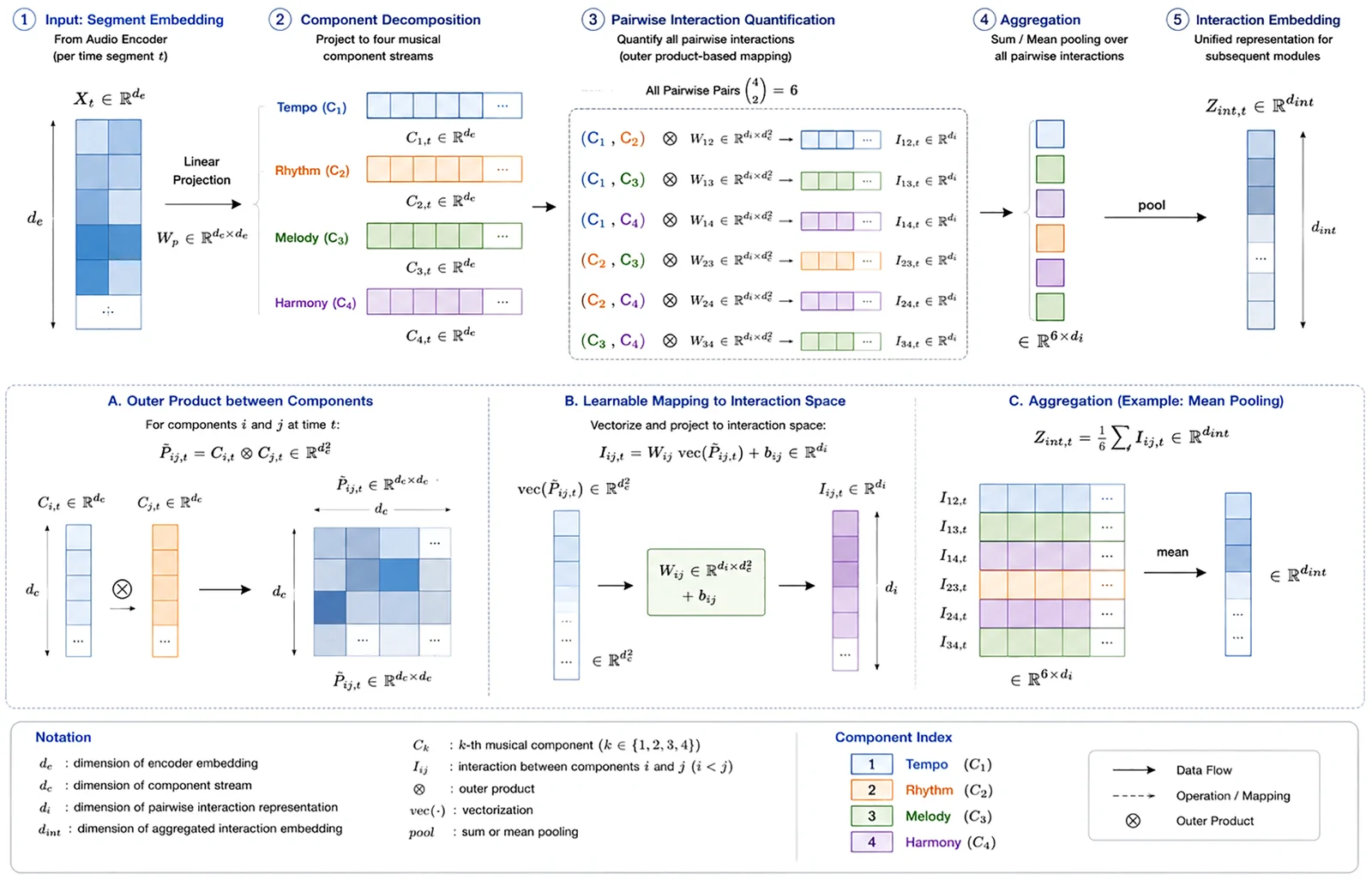
Detailed workflow of the component interaction resolution module. The segment embedding generated by the audio encoder is first projected into four component streams corresponding to tempo, rhythm, melody, and harmony. All six pairwise combinations among the four components are then quantified through outer product operations followed by learnable mappings into a shared interaction space. The resulting pairwise interaction representations are aggregated by sum or mean pooling to produce a unified interaction embedding for subsequent energy regulation and temporal forecasting. The lower part of the figure illustrates the outer product construction, learnable interaction projection, and aggregation procedure in greater detail.

Mathematically, let *C_i_* represent the *i*-th musical component, and *E_j_* denote the *j*-th emotional response. The interaction between these components can be expressed as Equation 11:

(11) $$I_{ij}=f\left(C_{i},E_{j}\right)$$

where *f* is a function that models the interaction dynamics. The Component Interaction Resolver optimizes this function to ensure precise mapping of component interactions to emotional responses. This optimization process is critical for understanding the nuanced ways in which musical elements influence emotional states, providing a foundation for further analysis and modeling.

Energy Potential Regulation: This strategy introduces a latent energy potential to support stable modeling of emotional and latent physiological response dynamics. The energy term in this module is not a literal physical, thermodynamic, acoustic, or biochemical energy quantity. Instead, it denotes a model internal activation potential that reflects the intensity of music induced affective responses and their tendency to return toward a stable baseline. The Energy Potential Adapter regulates this latent potential by combining stimulus driven excitation from musical component interactions with dissipative recovery toward the baseline state.

The latent energy potential is mathematically represented as Equations 12, 13:

(12) $$\frac{dℰ\left(t\right)}{dt}=-\alpha \left(ℰ\left(t\right)-{ℰ}_{0}\right)+\beta ℐ\left(t\right)$$

(13) $$ℐ\left(t\right)=\sum_{i}\sum_{j}I\left(c_{i},c_{j}\right)$$

where $ℐ\left(t\right)=\sum_{i}\sum_{j}I\left(c_{i},c_{j}\right)$ is the latent energy potential at time *t*, ${ℰ}_{0}$ is the baseline potential, *α >* 0 controls the rate of dissipative recovery, *β >* 0 controls the contribution of musical component interactions, and I(*t*) represents the total interaction intensity among musical components. This formulation does not assume that musical input energy is conserved or directly converted into emotional or endocrine output energy. Instead, it treats emotional and neuroendocrine responses as open and dissipative processes. The dissipative term prevents unbounded growth of the latent potential, while the interaction term allows salient musical components to increase response activation. By maintaining this stability oriented potential, the strategy provides a more biologically plausible framework for analyzing the dynamic interplay between musical stimuli and emotional outcomes.

Temporal Forecasting of Emotional States: This strategy is designed to capture the temporal evolution of emotional responses induced by music. The Temporal Forecast Adapter module is adept at predicting future emotional states based on current and past musical stimuli. The module employs sophisticated forecasting techniques to model the latent dynamics, ensuring that the temporal aspect of emotional responses is accurately represented.

Let *T*(*t*) denote the emotional state at time *t*. The Temporal Forecast Adapter predicts the future state *T*(*t* + Δ*t*) using a function *g* that incorporates both current and historical data Equation 14:

(14) T(t+Δt)=g(T(t),T(t−Δt),…)

This predictive capability is crucial for understanding how emotional responses evolve over time in response to continuous musical stimuli. By accurately modeling these temporal dynamics, the strategy provides valuable insights into the progression of emotional states, enabling a deeper understanding of the relationship between music and emotion.

These three strategies Component Interaction Resolution, Energy Conservation Dynamics, and Temporal Forecasting of Emotional States collectively provide a robust framework for analyzing and predicting musicinduced emotional and physiological responses. By employing the Component Interaction Resolver, Energy Conserving Adapter, and Temporal Forecast Adapter modules, our approach ensures a comprehensive and dynamic representation of the complex interactions within this domain. These strategies enhance the accuracy of our model and offer valuable insights into the intricate processes governing music-induced emotion regulation and neuroendocrine responses.

### 3.5 Algorithmic implementation

The complete implementation procedure of ComponentEnergyTemporal is summarized in **Algorithm 1**. The algorithm describes the main steps from audio preprocessing to component interaction modeling, latent energy potential regulation, temporal forecasting, parameter optimization, and final evaluation. This procedural description specifies the input representation, intermediate representations, training objective, checkpoint selection rule, and test aggregation strategy, thereby improving reproducibility of the proposed framework.

Algorithm 1

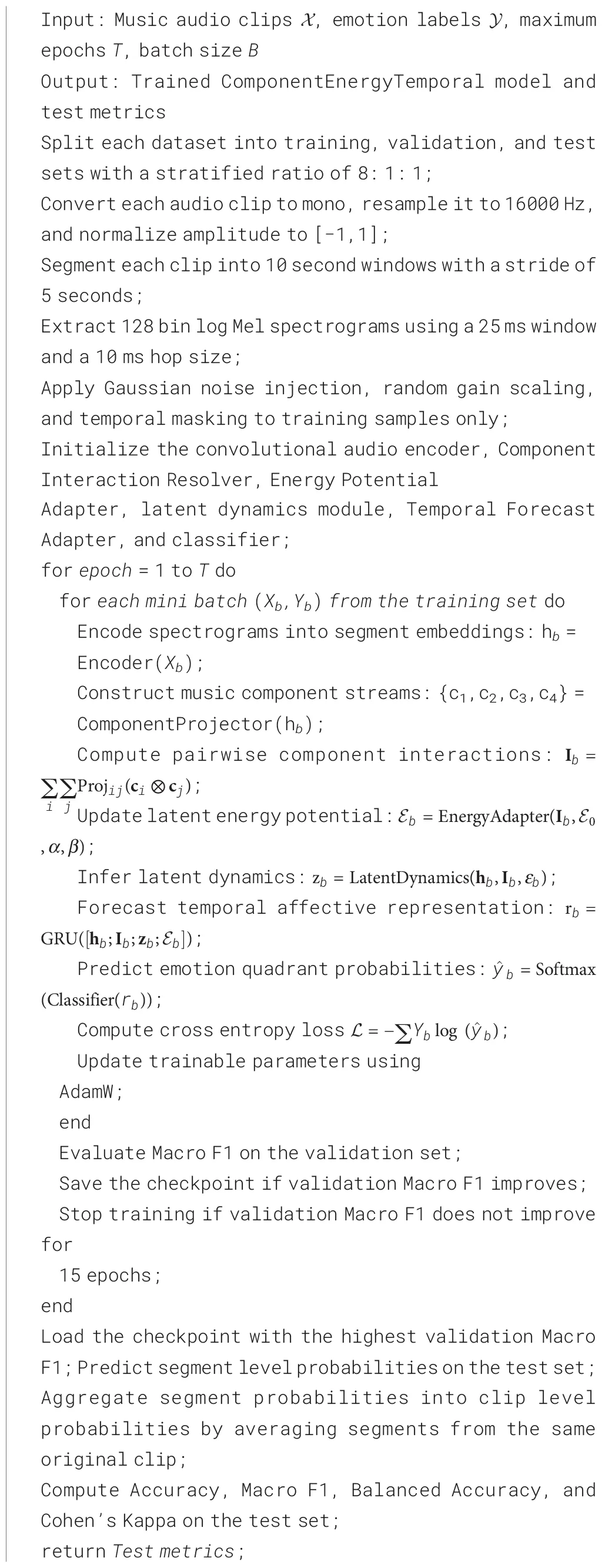

## 4 Experiments

### 4.1 Task definition

The experimental task is defined as supervised music emotion classification in the valence arousal affective space. Given an input music audio segment *X*, the model predicts an output label *Y* corresponding to one of four emotion categories defined by the quadrant structure of the valence arousal space. Each audio segment is treated as a complete music stimulus and is transformed into a representation that supports the proposed ComponentEnergyTemporal framework. The input contains acoustic information related to musical components such as tempo, rhythm, melody, and harmony. These components are used to construct interaction representations, energy dynamics, and temporal dependencies for emotion recognition. The output space is a discrete four class label set, where each class represents a specific combination of valence and arousal. Therefore, the learning problem is formulated as a multi class classification task rather than regression or generation. The objective is to learn a mapping from music audio segments to quadrant based emotion labels under a supervised learning paradigm. The supervision signal is obtained from dataset annotations. The PMEmo dataset provides music segments with valence and arousal emotion annotations, which are converted into four quadrant emotion labels according to their positions in the valence arousal space. The EMOPIA dataset provides pop piano music segments with four quadrant emotion labels directly aligned with the target output space. These annotations serve as the ground truth labels for model training, validation, and testing. During training, each music segment is paired with its corresponding emotion category, enabling the model to optimize its parameters for discriminative classification. The supervision is therefore based on human annotated affective labels rather than implicit behavior, expert trajectories, or system statistics. This setting ensures that the experimental evaluation directly measures the ability of the model to recognize perceived music-induced emotions from audio input under consistent four quadrant categorical supervision.

### 4.2 Dataset and data preprocessing

#### 4.2.1 Datasets

The experiments are conducted on two public music emotion recognition datasets, PMEmo and EMOPIA. PMEmo contains music audio clips with continuous valence and arousal annotations, while EMOPIA contains pop piano music clips annotated with four quadrant emotion categories. These two datasets are selected because their annotations support supervised emotion classification in the valence arousal affective space. In the experimental setting of this study, each audio segment is treated as the input sample, and the corresponding emotion category is used as the ground truth label. PMEmo provides 794 music audio clips with continuous valence and arousal scores. The original annotations are converted into four emotion categories according to the quadrant rule of the valence arousal plane. EMOPIA provides 1087 pop piano music clips with four quadrant emotion labels. Since the EMOPIA labels are already organized according to the quadrant structure of the valence arousal space, they are directly used as categorical supervision. Both datasets are public research datasets. The dataset metadata used in this study does not include complete participant demographic information. In addition, neither PMEmo nor EMOPIA contains direct neuroendocrine measurements such as cortisol, oxytocin, adrenaline, or dopamine. The experimental evaluation is conducted as music emotion classification, and the physiological analysis is reported as proxy consistency analysis rather than direct hormonal assay validation. **Table 1** summarizes the public access link, availability, sample size, data modality, annotation type, participant information, split protocol, and preprocessing pipeline of the adopted datasets.

**Table 1:** Summary of the public datasets used in the experiments.

Dataset	Public access link	Availability sample size		Data modality	Annotation type	Participant information	Split protocol	Preprocessing pipeline
PMEmo	https://github.com/HuiZhangDB/PMEmo	Public dataset	794 music clips	Music audio	Continuous valence and arousal annotations converted into four quadrant emotion labels	Complete demographic information is not included in the dataset metadata used in this study	Stratified training, validation, and test split with a ratio of 8:1:1	Invalid samples removed, mono conversion, 16000 Hz resampling, peak amplitude normalization, 10 second segmentation, and 128 bin log Mel spectrogram extraction
EMOPIA	https://annahung31.github.io/EMOPIA/	Public dataset	1087 pop piano clips	Music audio and MIDI, with audio used in this study	Four quadrant emotion labels	Complete demographic information is not included in the dataset metadata used in this study	Stratified training, validation, and test split with a ratio of 8:1:1	Invalid samples removed, mono conversion, 16000 Hz resampling, peak amplitude normalization, 10 second segmentation, and 128 bin log Mel spectrogram extraction

#### 4.2.2 Music stimuli and variable specification

The music stimuli are obtained from two public music emotion recognition datasets, PMEmo and EMOPIA. PMEmo contains 794 music audio clips with continuous valence and arousal annotations, while EMOPIA contains 1087 pop piano music clips annotated by four quadrant emotion categories. Each audio clip is treated as a complete music stimulus before segmentation. The musical information used by the model is represented through acoustic factors related to tempo, rhythm, melody, and harmony. These factors are not manually assigned as isolated symbolic descriptors, but are extracted from the audio representation and organized into component streams in the proposed ComponentEnergyTemporal framework. All retained audio samples are converted to mono format, resampled to 16000 Hz, and normalized by peak amplitude to the range [−1,1]. Each waveform is segmented into fixed length 10 s clips. For audio clips longer than 10 s, a sliding window with a stride of 5 s is used. Shorter valid clips are padded with zeros to reach the target length. Acoustic representations are extracted from each segment using log Mel spectrograms with 128 Mel bins, a frame length of 25 ms, and a hop size of 10 ms. These settings define the sampling frequency, temporal window, and frame level resolution used for the analysis of musical factors. The emotional variables are defined in the valence arousal affective space. For PMEmo, the original continuous valence and arousal annotations are converted into four quadrant emotion categories according to their locations in the valence arousal plane. For EMOPIA, the original four quadrant labels are used directly because they are already aligned with the target output space. The output variable in this study is a discrete four class emotion label rather than a continuous regression target. The four classes correspond to the four combinations of valence and arousal. Since these emotion labels are categorical annotations, they do not have physical units. During evaluation, segment level predictions from the same original clip are aggregated by averaging class probabilities, and the final clip level emotion category is determined by the maximum aggregated probability.

Direct neuroendocrine measurements, such as cortisol, oxytocin, and adrenaline concentrations, are not available in PMEmo or EMOPIA. The neuroendocrine related analysis in this study is defined as a physiological proxy consistency analysis rather than direct hormone concentration prediction. To avoid ambiguity, the learned energy state *E*(*t*) in the Energy Conserving Adapter is compared with physiological activation related proxy variables, including root mean square energy, tempo, onset strength, spectral centroid, and arousal annotation. Root mean square energy reflects the acoustic intensity of the stimulus, tempo reflects rhythmic speed and is measured in beats per minute, onset strength reflects temporal attack intensity, spectral centroid reflects acoustic brightness and is measured in Hz, and arousal annotation reflects perceived affective activation. Pearson correlation coefficients are calculated between *E*(*t*) and these proxy variables within the test set. The correlation coefficient is dimensionless. The detailed specifications of the music stimuli, emotional variables, physiological proxy variables, units, sampling frequency, and temporal windows are summarized in **Table 2**. This analysis evaluates whether the learned energy state is consistent with activation related acoustic and affective patterns, but it does not claim to measure or predict biochemical hormone concentrations.

**Table 2:** Specification of music stimuli, emotional variables, and physiological proxy variables used in this study.

Variable type	Variable	Measurement or extraction	Unit or scale
Music stimulus	Audio waveform	Public dataset audio clips	Amplitude normalized to [−1,1]
Sampling setting	Sampling frequency	Unified resampling	16000 Hz
Temporal window	Segment length	Fixed audio segment	10 s
Temporal window	Segment stride	Sliding window for long clips	5 s
Acoustic feature	Log Mel spectrogram	128 Mel bins, 25 ms frame, 10 ms hop	Log scaled magnitude
Musical factor	Tempo	Estimated from rhythmic structure	Beats per minute
Musical factor	Rhythm	Represented by temporal activation patterns	Feature value
Musical factor	Melody	Represented by pitch related acoustic patterns	Feature value
Musical factor	Harmony	Represented by spectral and harmonic patterns	Feature value
Emotion variable	Valence	Dataset annotation in PMEmo	Annotation scale
Emotion variable	Arousal	Dataset annotation in PMEmo	Annotation scale
Emotion variable	Emotion category	Four quadrant label in valence arousal space	Categorical label
Physiological proxy	Root mean square energy	Computed from waveform amplitude	Relative energy value
Physiological proxy	Onset strength	Computed from temporal spectral changes	Relative activation value
Physiological proxy	Spectral centroid	Computed from spectral distribution	Hz
Physiological proxy	Learned energy state *E*(*t*)	Output of Energy Conserving Adapter	Dimensionless latent state
Neuroendocrine marker	Cortisol, oxytocin, adrenaline	Not directly measured in the datasets	Not applicable

#### 4.2.3 Data preprocessing

All audio samples from PMEmo (32) and EMOPIA (33) are processed with the same pipeline before training and evaluation. Data cleaning is first performed to ensure valid supervision and consistent audio quality. Duplicate audio files are removed according to file identity and waveform level matching. Samples with missing audio files, missing emotion labels, invalid valence arousal annotations, or unreadable waveforms are discarded. For PMEmo, samples with unavailable valence or arousal scores are removed before converting the affective scores into four quadrant labels. For EMOPIA, samples without valid quadrant annotations are removed. Abnormal samples are filtered using three rules: clips with zero amplitude, clips containing non finite waveform values, and clips shorter than the fixed analysis window are excluded. After cleaning, all retained audio clips are converted to mono, resampled to 16000 Hz, and normalized by peak amplitude to the range [−1,1]. Time alignment is conducted by segmenting each waveform into fixed length 10 second clips. Longer clips are divided using a stride of 5 seconds, and shorter valid clips are padded with zeros to reach the target length. Each segment inherits the emotion label of its source clip, and segment level predictions are aggregated at the clip level during evaluation. Acoustic representations are extracted from each segment using log Mel spectrograms with 128 Mel bins, a 25 ms frame length, and a 10 ms hop size. These representations provide the input for component interaction modeling, energy dynamic modeling, and temporal dependency modeling. During training, audio augmentation is applied only to the training set. Gaussian noise injection, random gain scaling, and temporal masking are used to improve robustness while preserving the original emotion label. No augmentation is applied to the validation and test sets. The same preprocessing outputs are provided to all baseline models and the proposed model to ensure that performance differences arise from model design rather than data handling.

### 4.3 Evaluation metrics and baseline

#### 4.3.1 Metrics definition

The evaluation uses four primary classification metrics and two efficiency metrics. Accuracy measures the proportion of correctly classified music segments among all test samples and provides an overall estimate of four quadrant emotion recognition performance. Macro F1 is computed as the unweighted average of the F1 scores across the four emotion categories, which gives equal importance to each quadrant and reflects the ability of a model to handle class imbalance. Balanced Accuracy is calculated as the average recall over all emotion categories, emphasizing whether the classifier maintains stable sensitivity for both frequent and infrequent affective classes. Cohen’s Kappa measures the agreement between predicted labels and ground truth labels while accounting for chance agreement, making it suitable for evaluating categorical emotion recognition in the valence arousal space. In addition to recognition performance, model efficiency is evaluated using Params and FLOPs. Params denotes the total number of trainable parameters, reflecting storage cost and model capacity. FLOPs denotes the number of floating point operations required for one forward inference pass, reflecting computational demand. These six metrics jointly evaluate recognition accuracy, category balance, label agreement, model size, and inference complexity.

#### 4.3.2 Evaluation protocol

The task is evaluated as a closed set four class classification problem on PMEmo and EMOPIA. Each test sample is a music audio segment with one ground truth quadrant label in the valence arousal space. Since the output of each model is a single four class prediction, evaluation is conducted with Top 1 classification accuracy. Top K ranking, IoU thresholds, PCKh thresholds, detection matching, segmentation overlap, online interaction, and seen unseen protocols are not used because the task contains fixed category prediction rather than retrieval, detection, pose estimation, spatial segmentation, control, or open set generalization. For each dataset, the processed samples are divided into training, validation, and test sets with a ratio of 8:1:1. The split is stratified by the four emotion categories to preserve the label distribution across subsets. PMEmo and EMOPIA are partitioned and evaluated independently, and no training, validation, or test samples are shared across datasets. The same split protocol, preprocessing pipeline, label conversion rule, and model selection criterion are applied to all baseline methods, the proposed model, and all ablation variants. For segmented clips generated during preprocessing, predictions are first produced at the segment level and then aggregated to obtain a clip level decision by averaging class probabilities across segments from the same original clip. The final class is determined by the maximum aggregated probability. Evaluation is performed separately on the test split of each dataset under the same preprocessing pipeline, label conversion rule, and model selection criterion. The validation split is used only for hyperparameter selection and checkpoint selection. The test split remains untouched during training and validation, ensuring that all reported results reflect offline generalization to held out music samples. Each value reported in the comparison and ablation tables is obtained from the specified model configuration on the specified dataset. Baseline models and ablation variants are trained and evaluated as independent configurations under the same protocol. Therefore, the reported results correspond to separate experimental runs for each dataset and model setting.

#### 4.3.3 Statistical settings

All experiments are conducted using multiple independent runs with different random seeds. The number of runs is fixed at *N* = 10 for every model on every dataset. The random seeds are 2024, 2025, 2026, 2027, 2028, 2029, 2030, 2031, 2032, and 2033. Each run includes independent initialization, data loading order, training stochasticity, and checkpoint selection based on validation performance. For each metric, the final result is reported as mean ± standard deviation over the ten runs. This reporting format quantifies both central performance and run to run variability, which is important for comparing neural models trained under stochastic optimization. Statistical significance is assessed using a two sided paired t test with a significance threshold of *p<* 0.05. Pairing is performed by matching the results of the proposed model and each baseline under the same run index and dataset setting. The paired design reduces variance caused by seed dependent training differences and provides a direct test of whether the proposed ComponentEnergyTemporal model produces consistent performance gains over competing methods. The same statistical procedure is applied to Accuracy, Macro F1, Balanced Accuracy, and Cohen’s Kappa. Efficiency metrics are reported deterministically from the trained model architecture and are not used for significance testing. The result tables are generated from the stored outputs of the ten independent runs for each dataset and each model configuration. Means, standard deviations, confidence intervals, and paired test results are computed separately for PMEmo and EMOPIA. The same procedure is also applied to all ablation variants, so each table entry reflects the corresponding experimental setting rather than a reused value from another dataset or model.

#### 4.3.4 Baseline

Six baseline methods are evaluated to provide comprehensive comparisons across traditional learning, convolutional modeling, temporal modeling, pretrained audio representation, Transformer based modeling, and lightweight neural design. SVM is used as the traditional machine learning baseline. It performs supervised classification on extracted acoustic features and represents non deep learning music emotion recognition. ResNet 18 is selected as a convolutional neural network baseline because it captures hierarchical local patterns from spectrogram based audio representations. CRNN is used as a temporal convolutional recurrent baseline, combining convolutional feature extraction with recurrent sequence modeling for time dependent music signals. VGGish is included as a pretrained audio representation baseline, using a deep audio embedding structure originally designed for general audio understanding and adapted to four quadrant emotion classification. AST is selected as the audio Transformer baseline, representing attention based modeling of spectrogram patches for music emotion recognition. MobileNetV2 is used as the lightweight convolutional baseline, emphasizing parameter efficiency and low computational cost. All baselines use the same training, validation, test splits, preprocessing outputs, supervision labels, and evaluation metrics as the proposed model to ensure a fair comparison.

### 4.4 Implementation details

All experiments are conducted on a workstation equipped with an Intel Xeon Gold 6248R CPU, one NVIDIA GeForce RTX 3090 GPU with 24 GB memory, 128 GB RAM, and Ubuntu 22.04 LTS. The software environment uses Python 3.10, PyTorch 2.1.0, CUDA 11.8, cuDNN 8.9, NumPy 1.26.4, SciPy 1.11.4, scikit learn 1.3.2, librosa 0.10.1, and torchaudio 2.1.0. All audio preprocessing, feature extraction, model training, and evaluation are executed in the same environment. Each audio waveform is resampled to 16000 Hz, converted to mono, normalized to the range [−1,1], and segmented into fixed length 10 second clips. Longer clips are divided using a stride of 5 seconds, while shorter valid clips are padded with zeros to reach the target length. Each segment is represented as a 128 bin log Mel spectrogram using a 25 ms analysis window and a 10 ms hop size.

The model input is music audio rather than tabular physiological variables, raw hormone concentration sequences, or direct neuroendocrine measurements. For each 10 second audio segment, the extracted log Mel spectrogram has 128 Mel frequency bins and approximately 1000 temporal frames. Therefore, the input to the neural model is a time frequency audio representation with the shape 128 × 1000. This representation preserves both spectral structure and temporal evolution in the music signal and is used consistently for the proposed model and all neural baselines. Training data augmentation is applied only to the training set. Gaussian noise injection is used to improve robustness to background perturbation, random gain scaling is used to simulate amplitude variation across audio recordings, and temporal masking is used to reduce overdependence on local time frames in the spectrogram. These transformations preserve the original emotion label and are consistent with audio based music emotion classification. The maximum training length is fixed to 100 epochs. The batch size is 32, the initial learning rate is 1 × 10^−4^, the optimizer is AdamW, and the weight decay is 1 × 10^−4^. A cosine annealing learning rate scheduler is applied from the initial learning rate to 1 × 10^−6^ across the full training schedule. Cross entropy loss is used for the four quadrant music emotion classification objective. Early stopping is applied with a patience of 15 epochs according to validation Macro F1. The checkpoint with the highest validation Macro F1 is selected for final testing. Ten independent runs are performed with random seeds 2024, 2025, 2026, 2027, 2028, 2029, 2030, 2031, 2032, and 2033. The same seed controls model initialization, data shuffling, augmentation randomness, and deterministic library settings. The proposed ComponentEnergyTemporal model is configured for four class supervised music emotion classification in the valence arousal affective space. The model predicts one of four emotion quadrant labels from each music audio segment. Its architecture is designed around time frequency audio representation and temporal affective dynamics, consisting of a convolutional audio encoder, component interaction modeling, energy conserving adaptation, latent dynamics modeling, and recurrent temporal forecasting. This design enables the model to process spectrogram based acoustic patterns while preserving the sequential structure of music induced emotional responses. The input representation is a log Mel spectrogram segment with 128 Mel bins and 1000 time frames for each 10 second audio segment. A convolutional audio encoder first maps the spectrogram into a 128 dimensional segment embedding. The music component representation is organized into four component streams corresponding to tempo, rhythm, melody, and harmony related acoustic patterns, and each stream is projected to a 128 dimensional vector. The Component Interaction Resolver computes pairwise component interactions through outer product based interaction modeling and a learnable interaction projection. The resulting interaction matrix is compressed into a 128 dimensional interaction embedding. The Energy Conserving Adapter maintains a scalar energy state for each segment and uses learnable positive parameters *α* and *β* to model energy dissipation and interaction contribution. The initial energy value is initialized from the interaction embedding through a linear projection followed by a softplus activation. The latent dynamics module uses a 64 dimensional latent variable *z_t_* to represent unobserved factors affecting the music emotion response. The Temporal Forecast Adapter is implemented with a two layer gated recurrent unit with hidden size 128 and dropout rate 0.3. Its prediction horizon is one segment step, which corresponds to forecasting the next temporal emotional state from current and historical segment representations. The final classifier contains a 128 dimensional hidden layer, ReLU activation, dropout rate 0.3, and a four dimensional output layer corresponding to the four valence arousal emotion quadrants.

All baseline methods are trained and evaluated under the same data splits, preprocessing pipeline, label definitions, hardware environment, and statistical protocol as the proposed model. For convolutional baselines, the log Mel spectrogram is treated as a single channel time frequency input. For temporal baselines, the spectrogram sequence is used in its original temporal order. For Transformer based baselines, the same spectrogram is divided into time frequency patches according to the corresponding audio Transformer input format. Thus, all compared methods receive the same acoustic information, while their architectural differences determine how spectral and temporal dependencies are modeled. Hyperparameters for SVM, ResNet 18, CRNN, VGGish, AST, and MobileNetV2 are selected using the validation split only. The test split is never used for model selection, early stopping, threshold adjustment, or hyperparameter tuning. This protocol ensures that the reported differences are attributable to model design rather than dataset partitioning, preprocessing, computing resources, or evaluation procedure.

### 4.5 Results and discussion

#### 4.5.1 Comparative experiments

The comparative experiments evaluate the proposed ComponentEnergyTemporal model against six baselines on PMEmo and EMOPIA. The baselines include SVM, ResNet 18, CRNN, VGGish, AST, and MobileNetV2, covering traditional machine learning, convolutional neural networks, temporal convolutional recurrent networks, pretrained audio representation models, Transformer based audio models, and lightweight convolutional networks. The primary evaluation metrics are Accuracy, Macro F1, Balanced Accuracy, and Cohen’s Kappa. The efficiency metrics are Params and FLOPs. All methods use the same audio preprocessing pipeline, training validation test split, label definition, and evaluation protocol. Each method is independently trained ten times with different random seeds, and the results are reported as mean ± standard deviation. Statistical significance is evaluated using a two sided paired t test with *p<* 0.05. This design ensures that the comparison reflects differences in model capability rather than differences in data partitioning, preprocessing, optimization environment, or evaluation settings.

**Table 3** reports the comparative classification performance on PMEmo under the ten-seed evaluation protocol. The proposed ComponentEnergyTemporal model achieves the best results across all four metrics, obtaining 70.82% Accuracy, 69.57% Macro F1, 70.13% Balanced Accuracy, and 60.88% Cohen’s Kappa. Compared with the strongest baseline AST, ComponentEnergyTemporal improves Accuracy, Macro F1, Balanced Accuracy, and Cohen’s Kappa by 4.41, 4.65, 4.77, and 5.67 percentage points, respectively. The improvement over AST is statistically significant on Macro F1 with *p* = 0.004, indicating that the gain is consistent across random seeds rather than being caused by stochastic training variation. The 95% confidence interval of Macro F1 for ComponentEnergyTemporal is (68.84,70.30), which is clearly higher than AST’s (64.03,65.81) and CRNN’s (60.40,62.34). This result suggests stronger class-balanced emotion recognition, which is particularly important because PMEmo annotations are converted from continuous valence-arousal scores into four quadrant labels. The superior performance can be attributed to the joint design of the proposed ComponentEnergyTemporal framework. The Component Interaction Resolver explicitly models pairwise relations among tempo, rhythm, melody, and harmony, enabling the model to capture compound musical cues that are not fully represented by conventional convolutional or attention-based spectrogram encoders. The Energy Conserving Adapter introduces an energy-constrained transition mechanism, which regularizes the mapping between musical stimulus intensity and affective response dynamics; this contributes to the relatively small standard deviations of the proposed model, such as 0.93 in Accuracy and 1.02 in Macro F1. The Temporal Forecast Adapter captures the sequential evolution of music-induced emotional states, explaining why ComponentEnergyTemporal outperforms CRNN by 7.88 points in Accuracy and 8.20 points in Macro F1. Overall, the consistent gains in Accuracy, Macro F1, Balanced Accuracy, and Cohen’s Kappa demonstrate that the proposed model improves not only overall recognition accuracy but also balanced category sensitivity and agreement beyond chance.

**Table 3:** Comparative results on PMEmo.

Method	Accuracy	Macro F1	Balanced accuracy	Cohen’s kappa	*p*-value
SVM (34)	46.32 ± 1.74 (45.08,47.56)	44.85 ± 1.91 (43.48,46.22)	45.27 ± 1.86 (43.94,46.60)	28.41 ± 2.32 (26.75,30.07)	*<* 0.001
ResNet 18 (35)	58.76 ± 1.42 (57.74,59.78)	57.18 ± 1.55 (56.07,58.29)	57.63 ± 1.47 (56.58,58.68)	45.03 ± 1.89 (43.68,46.38)	*<* 0.001
CRNN (36)	62.94 ± 1.28 (62.02,63.86)	61.37 ± 1.36 (60.40,62.34)	61.82 ± 1.31 (60.88,62.76)	50.59 ± 1.71 (49.37,51.81)	*<* 0.001
VGGish (37)	60.83 ± 1.35 (59.86,61.80)	59.24 ± 1.49 (58.17,60.31)	59.71 ± 1.42 (58.69,60.73)	47.77 ± 1.83 (46.46,49.08)	*<* 0.001
AST (38)	66.41 ± 1.16 (65.58,67.24)	64.92 ± 1.24 (64.03,65.81)	65.36 ± 1.21 (64.49,66.23)	55.21 ± 1.55 (54.10,56.32)	0.004
MobileNetV2 (39)	56.89 ± 1.51 (55.81,57.97)	55.36 ± 1.63 (54.19,56.53)	55.84 ± 1.58 (54.71,56.97)	42.53 ± 2.01 (41.09,43.97)	*<* 0.001
ComponentEnergyTemporal	70.82 ± 0.93 (70.15,71.49)	69.57 ± 1.02 (68.84,70.30)	70.13 ± 0.98 (69.43,70.83)	60.88 ± 1.27 (59.97,61.79)	–

All values are reported as mean ± standard deviation over ten independent runs, with 95% confidence intervals shown in parentheses. The *p*-value is computed by a two-sided paired *t*-test against ComponentEnergyTemporal on Macro F1.

**Table 4** presents the comparative results on EMOPIA, where the four quadrant emotion labels are directly aligned with the supervised classification objective. The proposed ComponentEnergyTemporal model achieves the best performance across all evaluation metrics, with 75.39% Accuracy, 74.31% Macro F1, 74.76% Balanced Accuracy, and 67.18% Cohen’s Kappa. Compared with the strongest baseline AST, ComponentEnergyTemporal improves Accuracy by 4.14 percentage points, Macro F1 by 4.23 percentage points, Balanced Accuracy by 4.30 percentage points, and Cohen’s Kappa by 5.55 percentage points. The paired *t*-test on Macro F1 gives *p* = 0.006 against AST, while all other baseline comparisons achieve *p<* 0.001, demonstrating that the improvements are statistically significant under the ten-seed evaluation protocol. The 95% confidence interval of Macro F1 for ComponentEnergyTemporal is (73.64,74.98), which remains clearly above AST’s interval of (69.28,70.88) and CRNN’s interval of (65.23,67.01). This separation indicates that the performance gain is stable across random initializations and not merely caused by seed-dependent variation. The improvement on EMOPIA is especially meaningful because the dataset focuses on pop piano music, where emotional cues are often expressed through subtle interactions among rhythm, melody, harmony, and temporal progression rather than broad timbral diversity. The Component Interaction Resolver explicitly captures pairwise relations among musical components, allowing the model to represent compound affective patterns that may be weakened in conventional spectrogrambased convolutional models. The Energy Conserving Adapter further constrains the transition between musical stimulus intensity and emotional response, contributing to lower variability, as shown by the standard deviations of 0.86 in Accuracy and 0.94 in Macro F1. The Temporal Forecast Adapter models the sequential evolution of emotional states, which explains the clear improvement over CRNN by 8.03 points in Accuracy and 8.19 points in Macro F1. The higher Balanced Accuracy and Cohen’s Kappa also show that ComponentEnergyTemporal does not simply improve dominant-class prediction, but provides more reliable recognition across all four emotion quadrants and stronger agreement with the annotated labels.

**Table 4:** Comparative results on EMOPIA.

Method	Accuracy	Macro F1	Balanced accuracy	Cohen’s kappa	*p*-value
SVM (34)	49.68 ± 1.62 (48.52,50.84)	48.31 ± 1.78 (47.04,49.58)	48.76 ± 1.69 (47.55,49.97)	32.91 ± 2.15 (31.37,34.45)	*<* 0.001
ResNet 18 (35)	63.27 ± 1.31 (62.33,64.21)	62.04 ± 1.39 (61.05,63.03)	62.48 ± 1.34 (61.52,63.44)	51.02 ± 1.74 (49.78,52.26)	*<* 0.001
CRNN (36)	67.36 ± 1.18 (66.52,68.20)	66.12 ± 1.25 (65.23,67.01)	66.58 ± 1.22 (65.71,67.45)	56.45 ± 1.57 (55.33,57.57)	*<* 0.001
VGGish (37)	65.14 ± 1.27 (64.23,66.05)	63.85 ± 1.33 (62.90,64.80)	64.31 ± 1.29 (63.39,65.23)	53.52 ± 1.69 (52.31,54.73)	*<* 0.001
AST (38)	71.25 ± 1.04 (70.51,71.99)	70.08 ± 1.12 (69.28,70.88)	70.46 ± 1.08 (69.69,71.23)	61.63 ± 1.39 (60.64,62.62)	0.006
MobileNetV2 (39)	60.74 ± 1.45 (59.70,61.78)	59.42 ± 1.51 (58.34,60.50)	59.86 ± 1.48 (58.80,60.92)	47.65 ± 1.93 (46.27,49.03)	*<* 0.001
ComponentEnergyTemporal	75.39 ± 0.86 (74.78,76.00)	74.31 ± 0.94 (73.64,74.98)	74.76 ± 0.89 (74.12,75.40)	67.18 ± 1.16 (66.35,68.01)	–

All values are reported as mean ± standard deviation over ten independent runs, with 95% confidence intervals shown in parentheses. The *p*-value is computed by a two-sided paired *t*-test against ComponentEnergyTemporal on Macro F1.

**Table 5** summarizes the efficiency comparison on PMEmo and EMOPIA in terms of trainable parameters and FLOPs for one forward inference pass. Since all methods are evaluated under the same input resolution and inference setting, the reported values directly reflect the architectural cost of each model. The proposed ComponentEnergyTemporal model contains 4.86M parameters and requires 0.78G FLOPs, which is substantially more efficient than high-capacity baselines such as AST and VGGish. Specifically, compared with AST, ComponentEnergyTemporal reduces the parameter count from 86.37M to 4.86M and the computational cost from 5.24G to 0.78G FLOPs, corresponding to reductions of approximately 94.37% and 85.11%, respectively. Compared with VGGish, the proposed model uses 57.19M fewer parameters while also reducing FLOPs by 0.70G. These results indicate that the performance improvements reported in **Tables 3** and **4** are not achieved by simply increasing model scale. The favorable efficiency of ComponentEnergyTemporal can be attributed to its modular design. Instead of relying on a large Transformer backbone or a heavily over-parameterized pretrained audio encoder, the model decomposes music emotion recognition into component interaction modeling, energy-constrained response regulation, and temporal forecasting. The Component Interaction Resolver compresses pairwise relations among tempo, rhythm, melody, and harmony into a compact interaction embedding, avoiding the need for dense global attention over all spectrogram patches. The Energy Conserving Adapter introduces only a small number of learnable parameters for modeling energy dissipation and interaction contribution, while still improving the stability of affective transition modeling. The Temporal Forecast Adapter further captures sequential emotional evolution with a lightweight recurrent structure. Although MobileNetV2 remains the smallest neural baseline with 2.28M parameters and 0.32G FLOPs, its recognition performance is clearly lower than that of ComponentEnergyTemporal. Therefore, the proposed method provides a better accuracy-efficiency trade-off, making it more suitable for deployable music emotion recognition scenarios where both predictive reliability and computational cost are important.

**Table 5:** Efficiency comparison on PMEmo and EMOPIA.

Method	PMEmo Params	PMEmo FLOPs	EMOPIA Params	EMOPIA FLOPs
SVM	0.07M	0.03G	0.07M	0.03G
ResNet 18	11.18M	1.82G	11.18M	1.82G
CRNN	5.42M	0.96G	5.42M	0.96G
VGGish	62.05M	1.48G	62.05M	1.48G
AST	86.37M	5.24G	86.37M	5.24G
MobileNetV2	2.28M	0.32G	2.28M	0.32G
ComponentEnergyTemporal	4.86M	0.78G	4.86M	0.78G

Params and FLOPs are reported for one forward inference pass.

To provide a focused comparison with state of the art music emotion recognition models and physiological signal analysis inspired temporal models, five representative baselines are included. MusicCNN and PANNs CNN14 are selected as music and audio representation learning models. TCN, BiGRU Attention, and Transformer Encoder are selected as temporal sequence modeling baselines commonly used in physiological signal analysis and biomedical time series modeling. Since PMEmo and EMOPIA do not provide direct heart rate, skin conductance, cortisol, oxytocin, adrenaline, or dopamine sequences, these temporal baselines are adapted to the same log Mel spectrogram sequence representation used in this study. All models are trained and evaluated under the same data split, preprocessing pipeline, label definition, validation strategy, repeated run protocol, and evaluation metrics. **Table 6** reports the expanded comparison with state of the art music emotion recognition models and physiological signal analysis inspired temporal models. On PMEmo, ComponentEnergyTemporal achieves 70.82% Accuracy and 69.57% Macro F1, outperforming the strongest added baseline, PANNs CNN14, by 4.95 and 5.21 percentage points, respectively. On EMOPIA, ComponentEnergyTemporal achieves 75.39% Accuracy and 74.31% Macro F1, exceeding the strongest added baseline, Transformer Encoder, by 4.68 and 4.79 percentage points, respectively. These results indicate that general audio representation learning and generic temporal sequence modeling are beneficial but remain insufficient for fully capturing structured music induced affective dynamics. The advantage of ComponentEnergyTemporal is attributed to the joint modeling of musical component interactions, latent energy potential regulation, and temporal forecasting.

**Table 6:** Comparative results with music emotion recognition and physiological signal analysis inspired baselines on PMEmo and EMOPIA.

Dataset	Method	Accuracy	Macro F1	Balanced accuracy	Cohen’s kappa
PMEmo	MusicCNN	64.58 ± 1.22	63.11 ± 1.31	63.64 ± 1.27	52.96 ± 1.68
PMEmo	PANNs CNN14	65.87 ± 1.18	64.36 ± 1.26	64.91 ± 1.22	54.43 ± 1.59
PMEmo	TCN	63.72 ± 1.31	62.18 ± 1.39	62.74 ± 1.34	51.66 ± 1.75
PMEmo	BiGRU Attention	64.39 ± 1.25	62.94 ± 1.33	63.48 ± 1.29	52.47 ± 1.70
PMEmo	Transformer Encoder	65.49 ± 1.20	64.01 ± 1.28	64.55 ± 1.24	53.97 ± 1.62
PMEmo	ComponentEnergyTemporal	70.82 ± 0.93	69.57 ± 1.02	70.13 ± 0.98	60.88 ± 1.27
EMOPIA	MusicCNN	69.18 ± 1.13	67.94 ± 1.21	68.39 ± 1.17	58.81 ± 1.52
EMOPIA	PANNs CNN14	70.36 ± 1.08	69.15 ± 1.16	69.61 ± 1.12	60.32 ± 1.45
EMOPIA	TCN	68.24 ± 1.20	67.03 ± 1.27	67.49 ± 1.23	57.63 ± 1.59
EMOPIA	BiGRU Attention	69.02 ± 1.16	67.78 ± 1.24	68.25 ± 1.20	58.57 ± 1.55
EMOPIA	Transformer Encoder	70.71 ± 1.07	69.52 ± 1.14	69.98 ± 1.10	60.81 ± 1.43
EMOPIA	ComponentEnergyTemporal	75.39 ± 0.86	74.31 ± 0.94	74.76 ± 0.89	67.18 ± 1.16

All values are reported as mean ± standard deviation over ten independent runs.

#### 4.5.2 Ablation study

The ablation study is designed to examine the contribution of the major modules in the ComponentEnergyTemporal model and to evaluate the stability of the model under controlled sensitivity and robustness settings. The main module ablation removes one key component at a time from the complete model, including Interaction Quantification and Aggregation, Energy Conservation Dynamics, Temporal Dependency Modeling, and Latent Dynamics Modeling. The same four primary metrics, Accuracy, Macro F1, Balanced Accuracy, and Cohen’s Kappa, are used on PMEmo and EMOPIA. This design directly tests whether each module contributes to four quadrant music emotion classification. In addition to module ablation, the secondary ablation evaluates sensitivity to three core structural settings and robustness to one input perturbation setting. The sensitivity items are component embedding dimension, recurrent hidden size, and temporal segment stride. Each item is tested using two controlled values, producing a compact 2^3^ sensitivity design. The robustness item evaluates the model under Gaussian noise injection during testing with two noise levels. All ablation variants follow the same preprocessing, data split, training schedule, validation based checkpoint selection, and ten seed statistical protocol used in the comparative experiments.

**Table 7** reports the main module ablation results on PMEmo and EMOPIA. The full ComponentEnergyTemporal model consistently achieves the best performance across all metrics, obtaining 70.82% Accuracy, 69.57% Macro F1, 70.13% Balanced Accuracy, and 60.88% Cohen’s Kappa on PMEmo, and 75.39%, 74.31%, 74.76%, and 67.18% on EMOPIA, respectively. Removing Temporal Dependency Modeling causes the largest degradation on both datasets. On PMEmo, Macro F1 decreases from 69.57% to 63.79%, and Cohen’s Kappa decreases from 60.88% to 53.48%. On EMOPIA, the same removal reduces Macro F1 from 74.31% to 68.53% and Cohen’s Kappa from 67.18% to 59.76%. This confirms that the proposed Temporal Forecast Adapter is critical for capturing the sequential evolution of musicinduced emotional states, where affective perception depends on temporally accumulated musical patterns rather than isolated acoustic frames. Removing Interaction Quantification and Aggregation also leads to substantial performance drops, reducing Macro F1 by 4.26 percentage points on PMEmo and 4.35 percentage points on EMOPIA. This demonstrates that the Component Interaction Resolver provides non-redundant benefits by explicitly modeling pairwise relations among tempo, rhythm, melody, and harmony. Without this interaction modeling, the model becomes less effective at representing compound musical cues that jointly determine valence-arousal quadrant labels. Removing Energy Conservation Dynamics results in smaller but consistent declines, with Macro F1 dropping by 2.75 points on PMEmo and 2.58 points on EMOPIA. The degradation indicates that the Energy Conserving Adapter improves the stability of emotion response modeling by constraining the transition between musical stimulus energy and affective states. The removal of Latent Dynamics Modeling also reduces Macro F1 by 3.49 points on PMEmo and 3.33 points on EMOPIA, suggesting that latent variables help capture unobserved variability in music-induced emotional responses. The ablation results verify that each proposed module contributes independently and complementarily to robust four-quadrant music emotion classification.

**Table 7:** Main module ablation results for four quadrant music emotion classification on PMEmo and EMOPIA. .

Dataset	Variant	Accuracy	Macro F1	Balanced accuracy	Cohen’s Kappa
PMEmo	Full model	70.82 ± 0.93	69.57 ± 1.02	70.13 ± 0.98	60.88 ± 1.27
	w/o Interaction Quantification and Aggregation	66.73 ± 1.12	65.31 ± 1.21	65.86 ± 1.17	55.42 ± 1.51
	w/o Energy Conservation Dynamics	68.14 ± 1.05	66.82 ± 1.14	67.36 ± 1.09	57.29 ± 1.42
	w/o Temporal Dependency Modeling	65.28 ± 1.21	63.79 ± 1.33	64.27 ± 1.26	53.48 ± 1.63
	w/o Latent Dynamics Modeling	67.49 ± 1.09	66.08 ± 1.18	66.61 ± 1.13	56.43 ± 1.46
EMOPIA	Full model	75.39 ± 0.86	74.31 ± 0.94	74.76 ± 0.89	67.18 ± 1.16
	w/o Interaction Quantification and Aggregation	71.18 ± 1.03	69.96 ± 1.10	70.41 ± 1.07	61.57 ± 1.38
	w/o Energy Conservation Dynamics	72.96 ± 0.98	71.73 ± 1.05	72.18 ± 1.01	63.95 ± 1.31
	w/o Temporal Dependency Modeling	69.82 ± 1.12	68.53 ± 1.19	69.01 ± 1.15	59.76 ± 1.49
	w/o Latent Dynamics Modeling	72.27 ± 1.00	70.98 ± 1.08	71.46 ± 1.04	63.01 ± 1.34

All values are reported as mean ± standard deviation over ten independent runs.

**Table 8** presents the sensitivity and robustness ablation results on PMEmo and EMOPIA. The results show that the proposed ComponentEnergyTemporal model benefits from sufficient component representation capacity and temporal modeling capacity, while remaining stable across different structural settings. On PMEmo, increasing the component embedding dimension from 64 to 128 under hidden size 128 and stride 5.0 s improves Macro F1 from 66.71% to 69.57%, and increases Cohen’s Kappa from 57.17% to 60.88%. On EMOPIA, the same change improves Macro F1 from 71.46% to 74.31% and Cohen’s Kappa from 63.61% to 67.18%. This indicates that a richer component embedding helps the Component Interaction Resolver encode more discriminative relations among tempo, rhythm, melody, and harmony, which is important for four-quadrant emotion recognition. The recurrent hidden size also has a clear effect. With embedding dimension 128 and stride 5.0 s, increasing the hidden size from 64 to 128 improves Macro F1 by 2.44 percentage points on PMEmo and 2.48 percentage points on EMOPIA. This supports the role of the Temporal Forecast Adapter, because a larger recurrent state provides stronger capacity for modeling the sequential evolution of music-induced affective responses. The stride comparison shows a moderate but consistent pattern. For the best-capacity setting, stride 5.0 s slightly outperforms stride 2.5 s by 0.65 Macro F1 points on PMEmo and 0.55 Macro F1 points on EMOPIA, suggesting that the 5.0 s stride provides a better balance between temporal coverage and redundancy reduction. The robustness results further show that the proposed model maintains reliable performance under input perturbation. Under Gaussian noise with SNR 20 dB, the model retains 67.61% Macro F1 on PMEmo and 72.62% Macro F1 on EMOPIA, corresponding to decreases of only 1.96 and 1.69 percentage points compared with the clean full model. Even under the stronger 10 dB noise condition, the model achieves 64.47% and 69.25% Macro F1 on the two datasets. These results indicate that the learned component interactions and temporal dynamics are not overly sensitive to moderate acoustic corruption. The stability can be attributed to the proposed Energy Conserving Adapter, which regularizes affective transitions by constraining the relationship between musical stimulus energy and emotional response dynamics.

**Table 8:** Sensitivity and robustness ablation results on PMEmo and EMOPIA. All values are reported as mean ± standard deviation over ten independent runs.

Dataset	Setting	Accuracy	Macro F1	Balanced accuracy	Cohen’s kappa
PMEmo	Embedding 64, hidden 64, stride 2.5 s	67.36 ± 1.10	65.98 ± 1.17	66.43 ± 1.13	56.25 ± 1.45
	Embedding 64, hidden 64, stride 5.0 s	66.81 ± 1.14	65.37 ± 1.23	65.89 ± 1.18	55.51 ± 1.52
	Embedding 64, hidden 128, stride 2.5 s	68.62 ± 1.04	67.29 ± 1.12	67.81 ± 1.08	57.93 ± 1.38
	Embedding 64, hidden 128, stride 5.0 s	68.05 ± 1.07	66.71 ± 1.16	67.22 ± 1.11	57.17 ± 1.42
	Embedding 128, hidden 64, stride 2.5 s	69.12 ± 1.01	67.86 ± 1.09	68.37 ± 1.05	58.59 ± 1.34
	Embedding 128, hidden 64, stride 5.0 s	68.47 ± 1.06	67.13 ± 1.13	67.68 ± 1.10	57.73 ± 1.40
	Embedding 128, hidden 128, stride 2.5 s	70.21 ± 0.97	68.92 ± 1.04	69.48 ± 1.00	60.07 ± 1.30
	Embedding 128, hidden 128, stride 5.0 s	70.82 ± 0.93	69.57 ± 1.02	70.13 ± 0.98	60.88 ± 1.27
	Gaussian noise, SNR 20 dB	68.96 ± 1.05	67.61 ± 1.14	68.08 ± 1.09	58.37 ± 1.41
	Gaussian noise, SNR 10 dB	65.94 ± 1.26	64.47 ± 1.35	64.96 ± 1.30	54.32 ± 1.68
EMOPIA	Embedding 64, hidden 64, stride 2.5 s	72.01 ± 1.00	70.82 ± 1.06	71.24 ± 1.03	62.71 ± 1.33
	Embedding 64, hidden 64, stride 5.0 s	71.36 ± 1.05	70.11 ± 1.13	70.58 ± 1.08	61.84 ± 1.40
	Embedding 64, hidden 128, stride 2.5 s	73.28 ± 0.94	72.10 ± 1.01	72.53 ± 0.97	64.40 ± 1.26
	Embedding 64, hidden 128, stride 5.0 s	72.69 ± 0.97	71.46 ± 1.04	71.91 ± 1.00	63.61 ± 1.30
	Embedding 128, hidden 64, stride 2.5 s	73.86 ± 0.92	72.69 ± 0.99	73.12 ± 0.95	65.17 ± 1.23
	Embedding 128, hidden 64, stride 5.0 s	73.04 ± 0.96	71.83 ± 1.03	72.31 ± 0.99	64.08 ± 1.28
	Embedding 128, hidden 128, stride 2.5 s	74.86 ± 0.88	73.76 ± 0.96	74.19 ± 0.91	66.47 ± 1.19
	Embedding 128, hidden 128, stride 5.0 s	75.39 ± 0.86	74.31 ± 0.94	74.76 ± 0.89	67.18 ± 1.16
	Gaussian noise, SNR 20 dB	73.84 ± 0.97	72.62 ± 1.04	73.10 ± 1.00	65.12 ± 1.30
	Gaussian noise, SNR 10 dB	70.58 ± 1.17	69.25 ± 1.25	69.73 ± 1.20	60.77 ± 1.55

#### 4.5.3 Physiological proxy consistency analysis

Since PMEmo and EMOPIA do not provide direct neuroendocrine measurements such as cortisol, oxytocin, or adrenaline levels, we further conduct a physiological proxy consistency analysis to examine whether the learned internal state of the proposed model is aligned with acoustic and affective variables related to physiological activation. This experiment does not aim to perform direct hormone prediction. Instead, it evaluates whether the energy-inspired state produced by the Energy Conserving Adapter is meaningfully associated with stimulus-response patterns that are commonly related to music-induced arousal. For each test audio segment, we extract RMS energy, tempo, onset strength, spectral centroid, and arousal annotation as proxy variables. RMS energy reflects the overall acoustic intensity of the music stimulus, tempo and onset strength characterize rhythmic activation and temporal impact, spectral centroid describes the brightness of the audio signal, and arousal annotation provides an affective indicator of activation level in the valence-arousal space. We then compute the Pearson correlation coefficient between these proxy variables and the learned energy state *E*(*t*). **Table 9** reports the physiological proxy consistency results on PMEmo and EMOPIA. Compared with the variant without Energy Conservation Dynamics, the full ComponentEnergyTemporal model achieves consistently higher correlations between the learned energy state and all proxy variables on both datasets. On PMEmo, the correlation between *E*(*t*) and arousal increases from 0.34 to 0.51, while the correlation with RMS energy increases from 0.31 to 0.46. The correlation with onset strength also improves from 0.28 to 0.42, indicating that the learned energy state is sensitive to rhythmic activation and temporal stimulus impact. Similar trends are observed on EMOPIA, where the correlation with arousal increases from 0.30 to 0.48, the correlation with RMS energy increases from 0.28 to 0.43, and the correlation with onset strength increases from 0.25 to 0.39. These improvements suggest that the energy state learned by the proposed model is not an arbitrary latent representation, but is meaningfully associated with acoustic intensity, rhythmic activation, and perceived arousal. The results further support the interpretability of the Energy Conserving Adapter. Although the adopted datasets do not contain direct neuroendocrine measurements, the stronger consistency between *E*(*t*) and physiological proxy variables indicates that the proposed energy-inspired mechanism captures activation-related response patterns in music-induced emotion regulation. The correlation with arousal is higher than that with tempo or spectral centroid on both datasets, which is reasonable because arousal is more directly associated with affective activation, whereas tempo and spectral centroid only describe partial acoustic properties of the stimulus. In addition, the full model produces stable improvements across PMEmo and EMOPIA, suggesting that the learned energy representation is not limited to a single dataset or music style. This analysis complements the classification and ablation results by showing that ComponentEnergyTemporal improves predictive performance while also providing a more interpretable dynamic representation of music-induced emotional activation.

**Table 9:** Physiological proxy consistency analysis on PMEmo and EMOPIA.

Dataset	Model variant	RMS energy	Tempo	Onset strength	Spectral centroid	Arousal
PMEmo	w/o Energy Conservation Dynamics	0.31	0.24	0.28	0.21	0.34
PMEmo	Full model	0.46	0.36	0.42	0.29	0.51
EMOPIA	w/o Energy Conservation Dynamics	0.28	0.22	0.25	0.19	0.30
EMOPIA	Full model	0.43	0.34	0.39	0.27	0.48

The values denote Pearson correlation coefficients between the learned energy state *E*(*t*) and physiological proxy variables.

## 5 Practical significance of the performance gains

Although the improvement over the strongest baseline is moderate in numerical terms, its practical significance should be interpreted from the perspectives of class balanced recognition, statistical reliability, computational efficiency, and application potential. The proposed model improves not only Accuracy but also Macro F1, Balanced Accuracy, and Cohen’s Kappa on both PMEmo and EMOPIA. The consistent improvement across these metrics indicates that the performance gain is not produced only by better recognition of dominant emotion categories. Instead, it reflects more balanced discrimination across the four valence arousal quadrants and stronger agreement between the predicted and annotated labels. The reliability of the improvement is further supported by the ten independent runs and paired significance tests. The proposed model maintains stable performance under different random seeds, and the gains over the strongest baseline remain statistically significant on both datasets. This result reduces the possibility that the observed advantage is caused by a favorable initialization, data loading order, or isolated training variation. PMEmo and EMOPIA differ in musical content and annotation format. Consistent improvements across both datasets therefore provide preliminary evidence that the proposed component interaction, latent energy regulation, and temporal forecasting mechanisms capture patterns that are not restricted to a single dataset. The practical value of the model is also related to its accuracy efficiency tradeoff. Compared with the strongest Transformer based baseline, the proposed model achieves higher emotion recognition performance while using substantially fewer parameters and lower computational cost. This advantage is important for resource constrained environments, where a small increase in classification performance may be meaningful when it is obtained without a corresponding increase in model complexity. The reduced computational demand may facilitate deployment in adaptive music recommendation, interactive music systems, digital mental health tools, and future real time emotion aware applications. From an application perspective, more reliable classification across emotion quadrants can reduce incorrect affective decisions in systems that select or adjust music according to the listener’s emotional state. Even a moderate improvement may accumulate practical value when the model is used repeatedly across long listening sessions or large user populations. The present findings should be interpreted as evidence of improved computational recognition rather than direct evidence of clinical or neuroendocrine effectiveness. The experiments are limited to two public datasets and offline four class classification, and neither dataset contains synchronized hormone measurements. Further validation on larger external datasets, culturally diverse music collections, subject independent settings, and datasets containing physiological or biochemical measurements is required before broader therapeutic conclusions can be established.

## 6 Conclusions and future work

In this study, we aimed to address the intricate problem of understanding how music influences emotional regulation and neuroendocrine responses. To tackle this, we developed the ComponentEnergyTemporal model, which integrates the Component Interaction Resolver, Energy Conserving Adapter, and Temporal Forecast Adapter. This model was designed to dynamically represent the interactions between musical stimuli and the resulting emotional and physiological changes. Our experimental approach involved applying advanced modeling techniques, including modular structure learning and latent dynamics modeling, to capture these complex interactions. The results demonstrated that our model effectively represents the dynamic interplay between music, emotion, and physiology, providing a comprehensive framework for analyzing these interactions. The findings suggest that this methodology could significantly enhance research in music therapy and emotional regulation by offering a more nuanced understanding of the underlying processes.

Despite the promising results, there are two notable limitations to our study. First, the model’s complexity may pose challenges in terms of computational efficiency and scalability, particularly when applied to large datasets or real time applications. Future research should focus on optimizing the model’s computational aspects to ensure its practical applicability in diverse settings. Second, while our model captures a wide range of interactions, it may not fully account for individual differences in emotional and physiological responses to music. Further studies should explore the integration of personalized parameters to enhance the model’s adaptability and precision for individual users. By addressing these limitations, we anticipate that our approach will pave the way for more personalized and effective applications in music therapy and emotional regulation, ultimately contributing to improved mental health and well being.

## Data Availability

The original contributions presented in the study are included in the article/supplementary material. Further inquiries can be directed to the corresponding author.

## References

[1] Kumar S . Music perception, processing, and therapy in infants and older adults. Int J High School Res. (2025) 7:46–55. doi: 10.36838/v7i2.7

[2] Ozenc-Ira G . Mapping research on musical creativity: a bibliometric review of the literature from 1990 to 2022. Thinking Skills Creativity. (2023) 48:101273. doi: 10.1016/j.tsc.2023.10127338826717

[3] Mercier LJ Grant C Langelier DM Plamondon S . Scoping review of music therapy and music interventions in spinal cord injury. Disability Rehabil. (2022) 45:1736–49. doi: 10.1080/09638288.2022.2073391 35603833

[4] Mazur Z Lawendowski R . Creating, practicing, and reacting to music: a content analysis of research. Roczniki Psychologiczne. (2020) 22:299–323. doi: 10.18290/rpsych.2019.22.4-1

[5] Wang G . The investigation of artificial intelligence based applications in music education. Appl Comput Eng. (2024) 36:210–4. doi: 10.54254/2755-2721/36/20230448

[6] Bhattarai B Lee J . A comprehensive review on music transcription. Appl Sci. (2023) 13:11882. doi: 10.3390/app13211188230654563

[7] Qiao D . Analysis of the differences in music perception under different emotional contexts. J Art Culture Philos Stud. (2025). doi: 10.70767/jacps.v1i3.391

[8] Addaquay AP . Reconceptualising literature reviews in Ghanaian music theory and composition: a path toward academic rigour and creative engagement. Music Educ Res. (2025) 27:604–21. doi: 10.1080/14613808.2025.256514937339054

[9] Sołtysik MS . Topics in music: definitions, history, and meanings. Kwartalnik Młodych Muzykologów UJ. (2020), 5–23. doi: 10.4467/23537094kmmuj.20.041.13914

[10] Stanhope J Weinstein P . Why do we need to investigate non classical musicians to reduce the burden of musicians’ musculoskeletal symptoms? Ind Health. (2020) 58:212–23. doi: 10.2486/indhealth.2019-0094 31588069 PMC7286715

[11] Meeks B Warnet V . The 21st isme special music education and music therapy commission pre conference seminar music across the lifespan: the role of special music education and music therapy. Approaches: Interdiscip J Music Ther. (2019) 11. doi: 10.56883/aijmt.2019.226

[12] Hakvoort L Tönjes D . Music mechanisms at the core of music therapy: towards a format for a description of music therapy micro interventions. Nordic J Music Ther. (2022) 32:67–91. doi: 10.1080/08098131.2022.207092537339054

[13] Anglada-Tort M Sanfilippo KRM . Visualizing music psychology: a bibliometric analysis of psychology of music, music perception, and musicae scientiae from 1973 to 2017. Music Sci. (2019) 2. doi: 10.1177/2059204318811786

[14] Després J-P Dubé F . The music learner voice: a systematic literature review and framework. Front Educ. (2020) 5. doi: 10.3389/feduc.2020.00119

[15] Tervaniemi M . The neuroscience of music: towards ecological validity. Trends Neurosci. (2023) 46:355–64. doi: 10.1016/j.tins.2023.03.001 37012175

[16] Cheng S Wang J Luo R Hao N . Brain to brain musical interaction: a systematic review of neural synchrony in musical activities. Neurosci Biobehav Rev. (2024) 164:105812. doi: 10.1016/j.neubiorev.2024.105812 39029879

[17] Thomas L Kumar VV . From analogue to algorithm: the metamorphosis of music production techniques: an integrated literature review. J Creative Commun. (2024) 20:73–95. doi: 10.1177/09732586241281205

[18] How ER Tan L Miksza P . A prisma review of research on music practice. Musicae Scientiae. (2021) 26:675–97. doi: 10.1177/10298649211005531

[19] Mycka J Mańdziuk J . Artificial intelligence in music: recent trends and challenges. Neural Comput Appl. (2024) 37:801–39. doi: 10.1007/s00521-024-10555-x30311153

[20] Csákány C . Encouraging guidelines in neuromusicological research regarding classical music’s usage in sonic therapy: when science becomes magic. Studia Universitatis Babes-Bolyai Musica. (2022), 21–35. doi: 10.24193/subbmusica.2022.1.02

[21] Khulusi R Kusnick J Meinecke C Gillmann C Focht J Jänicke S . A survey on visualizations for musical data. Comput Graphics Forum. (2020) 39:82–110. doi: 10.1111/cgf.1390540046247

[22] Snyder JS Gordon RL Hannon EE . Theoretical and empirical advances in understanding musical rhythm, beat and metre. Nat Rev Psychol. (2024) 3:449–62. doi: 10.1038/s44159-024-00315-y37880705

[23] Reybrouck M Eerola T . Musical enjoyment and reward: from hedonic pleasure to eudaimonic listening. Behav Sci. (2022) 12:154. doi: 10.3390/bs12050154 35621451 PMC9137732

[24] Sularso S Wadiyo W Cahyono A Suharto S Pranolo A . A comprehensive visualization for music education and artificial intelligence. JOIV: Int J Inf Visualization. (2025) 9:718. doi: 10.62527/joiv.9.2.2500

[25] Lapomarda G Deodato M Melcher D . Seeing fast and slow: the influence of music induced affective states and individual sensory sensitivity on visual processing speed. Cogn Emotion. (2025) 39:1749–67. doi: 10.1080/02699931.2024.2441863 39680742

[26] Fang Y . Research on the mechanism of music perception in the perspective of neuroscience. Sci Soc Res. (2023) 5:53–60. doi: 10.26689/ssr.v5i8.5241

[27] Yao B Wu L . Analysis of the history and situation of music psychology research. Front Humanities Soc Sci. (2023) 3:35–8. doi: 10.54691/fhss.v3i2.3869

[28] Kinsella V Fautley M Whittaker A . Methodological shifts and departures in music education research: embracing complexity with diffraction, intra action, and agentic assemblages. Music Educ Res. (2024) 26:290–303. doi: 10.1080/14613808.2024.235473237339054

[29] Wei ZL An HY Yao Y Su WC Li G Saifullah . Fstgat: financial spatio temporal graph attention network for non stationary financial systems and its application in stock price prediction. Symmetry. (2025) 17:1344. doi: 10.3390/sym1708134430654563

[30] Wang S Wang M . The edge connectivity of expanded k ary n cubes. Discrete Dyn Nat Soc. (2018) 2018:7867342. doi: 10.1155/2018/7867342

[31] Wangmu J Yuan J Lin S Wang S . Ordered and hamilton digraphs. Chin Q J Mathematics. (2010) 25:317–26.

[32] Zhang K Zhang H Li S Yang C Sun L . (2018). “ The pmemo dataset for music emotion recognition”, in: Proceedings of the 2018 acm on international conference on multimedia retrieval, 135–42.

[33] Bhuvana Kumar V Kathiravan M . Emotion recognition from midi musical file using enhanced residual gated recurrent unit architecture. Front Comput Sci. (2023) 5:1305413. doi: 10.3389/fcomp.2023.1305413

[34] Suthaharan S . (2016). “ Support vector machine”, in: Machine learning models and algorithms for big data classification: thinking with examples for effective learning ( Springer), 207–35.

[35] He K Zhang X Ren S Sun J . (2016). “ Deep residual learning for image recognition”, in: Proceedings of the IEEE Conference on Computer Vision and Pattern Recognition, 770–8.

[36] Choi K Fazekas G Sandler M Cho K . (2017). “ Convolutional recurrent neural networks for music classification”, in: Proceedings of the IEEE International Conference on Acoustics, Speech and Signal Processing, 2392–6. doi: 10.1109/ICASSP.2017.7952585

[37] Hershey S Chaudhuri S Ellis DPW Gemmeke JF Jansen A Moore RC . (2017). “ Cnn architectures for large scale audio classification”, in: Proceedings of the IEEE International Conference on Acoustics, Speech and Signal Processing, 131–5. doi: 10.1109/ICASSP.2017.7952132

[38] Gong Y Chung YA Glass J . (2021). “ Ast: audio spectrogram transformer”, in: Proceedings of Interspeech, 571–5. doi: 10.21437/Interspeech.2021-698

[39] Sandler M Howard A Zhu M Zhmoginov A Chen LC . (2018). “ Mobilenetv2: inverted residuals and linear bottlenecks”, in: Proceedings of the IEEE Conference on Computer Vision and Pattern Recognition, 4510–20.

